# 67,000 years of coastal engagement at Panga ya Saidi, eastern Africa

**DOI:** 10.1371/journal.pone.0256761

**Published:** 2021-08-26

**Authors:** Patrick Faulkner, Jennifer M. Miller, Eréndira M. Quintana Morales, Alison Crowther, Ceri Shipton, Emmanuel Ndiema, Nicole Boivin, Michael D. Petraglia

**Affiliations:** 1 Department of Archaeology, The University of Sydney, Sydney, New South Wales, Australia; 2 Department of Archaeology, Max Planck Institute for the Science of Human History, Jena, Germany; 3 Department of Anthropology, University of California Santa Cruz, Santa Cruz, California, United States of America; 4 School of Social Science, The University of Queensland, Brisbane, Queensland, Australia; 5 Institute of Archaeology, University College London, London, United Kingdom; 6 Centre of Excellence for Australian Biodiversity and Heritage, College of Asia and the Pacific, Australian National University, Canberra, Australian Capital Territory, Australia; 7 Department of Earth Sciences, Archaeology Section, National Museums of Kenya, Nairobi, Kenya; 8 Department of Anthropology, National Museum of Natural History, Smithsonian Institution, Washington, DC, United States of America; 9 Department of Anthropology and Archaeology, University of Calgary, Calgary, Canada; 10 Australian Research Centre for Human Evolution (ARCHE), Griffith University, Brisbane, Australia; University at Buffalo - The State University of New York, UNITED STATES

## Abstract

The antiquity and nature of coastal resource procurement is central to understanding human evolution and adaptations to complex environments. It has become increasingly apparent in global archaeological studies that the timing, characteristics, and trajectories of coastal resource use are highly variable. Within Africa, discussions of these issues have largely been based on the archaeological record from the south and northeast of the continent, with little evidence from eastern coastal areas leaving significant spatial and temporal gaps in our knowledge. Here, we present data from Panga ya Saidi, a limestone cave complex located 15 km from the modern Kenyan coast, which represents the first long-term sequence of coastal engagement from eastern Africa. Rather than attempting to distinguish between coastal resource use and coastal adaptations, we focus on coastal engagement as a means of characterising human relationships with marine environments and resources from this inland location. We use aquatic mollusc data spanning the past 67,000 years to document shifts in the acquisition, transportation, and discard of these materials, to better understand long-term trends in coastal engagement. Our results show pulses of coastal engagement beginning with low-intensity symbolism, and culminating in the consistent low-level transport of marine and freshwater food resources, emphasising a diverse relationship through time. Panga ya Saidi has the oldest stratified evidence of marine engagement in eastern Africa, and is the only site in Africa which documents coastal resources from the Late Pleistocene through the Holocene, highlighting the potential archaeological importance of peri-coastal sites to debates about marine resource relationships.

## Introduction

The way in which people engaged with coastlines and coastal resources is a central issue in Palaeolithic archaeology. Marine habitats and resources have been touted as essential ingredients in the evolution of our species (e.g., [1, 2]), crucial to the movement of modern humans around the globe (e.g., [3, 4]), and evidence of adaptation to diverse environments (e.g., [5–7]). As with many aspects of behavioural evolution, attention has been focused on Africa for early evidence of coastal engagement. The timing, nature, and trajectories of coastal resource use and/or adaptations appear to be highly variable through time and space [8]. However, most African sites with marine evidence are found at the northern and southern extremes of the continent, and only capture short pulses of occupation. This leaves a significant gap in our understanding of the antiquity of coastal resource use in eastern Africa. It is therefore crucial to trace the development of these behaviours in eastern Africa, to appreciate the influence of marine resources on economy, landscape use, and symbolism.

In archaeological research, the terms *coastal resource use* and *coastal adaptation* carry specific connotations [1, 9], though there is no widespread agreement on when the transition between the two occurs. Coastal resource use can be defined as the systematic and recurrent exploitation of marine resources, but where lifeways are not necessarily transformed. On the other hand, coastal adaptation occurs with the effective, repeated, and dominant exploitation of marine environments and the intertidal zone (e.g., [1, 2, 10]). This intense relationship shapes all aspects of a culture including diet, technology, mobility systems, and socio-economic organization. These concepts have been applied to dense shell midden sites in coastal South Africa, but even there the onset of coastal adaptations have been variably assigned to the Middle Stone Age (110 kya, MIS 5) [1, 11] or to the Later Holocene (3.5 kya) [9]. The broader definition of coastal adaptation espoused by Will and colleagues [11, 12] may provide a way forward, focusing on a range of complex behavioural traits that incorporates coastal landscape occupation and use of marine resources. In this view, coastal adaptations would therefore consist of settlement systems that included coastal and near-coastal areas on a regular/planned basis, as well as the systematic and regular use and transportation of marine resources. However, there is no clear methodology to apply these terms to peri-coastal sites, and no suitable term for sites with sporadic, intermittent, or tangential contact with marine resources.

Here, we employ the term coastal *engagement* as a broad term for any relationship with marine environments. It can encompass the full spectrum of intensity from sporadic, indirect contact with the coast to fully developed maritime or seafaring adaptations, but importantly, this term can include subtle evidence for marine resource use. This may be irregular or unsystematic, and therefore does not fulfill the criteria outlined above for resource use, but it must be quantified and described in a consistent way to understand the variability of human-coast interactions, especially in eastern Africa. This approach is important in the context of recent discussions around the adaptive plasticity of our species to diverse conditions [13, 14], where characterising the nature and trajectory of human behaviour and ecosystem interactions over the long-term is crucial. We further suggest that engagement is an appropriate term for peri-coastal sites where the mechanisms of coastal contact are unclear, as seen at MSA sites such as Varsche Rivier 003, Diepkloof Rock Shelter, and Sibudu Cave [15–17], as these sites in South Africa all contain marine fauna and are 10 km or more from the current coast. These near-coastal sites do not have dense, shell supported midden deposits, but can provide unique insights into the long-distance transport and discard of marine resources.

**Fig 1:**
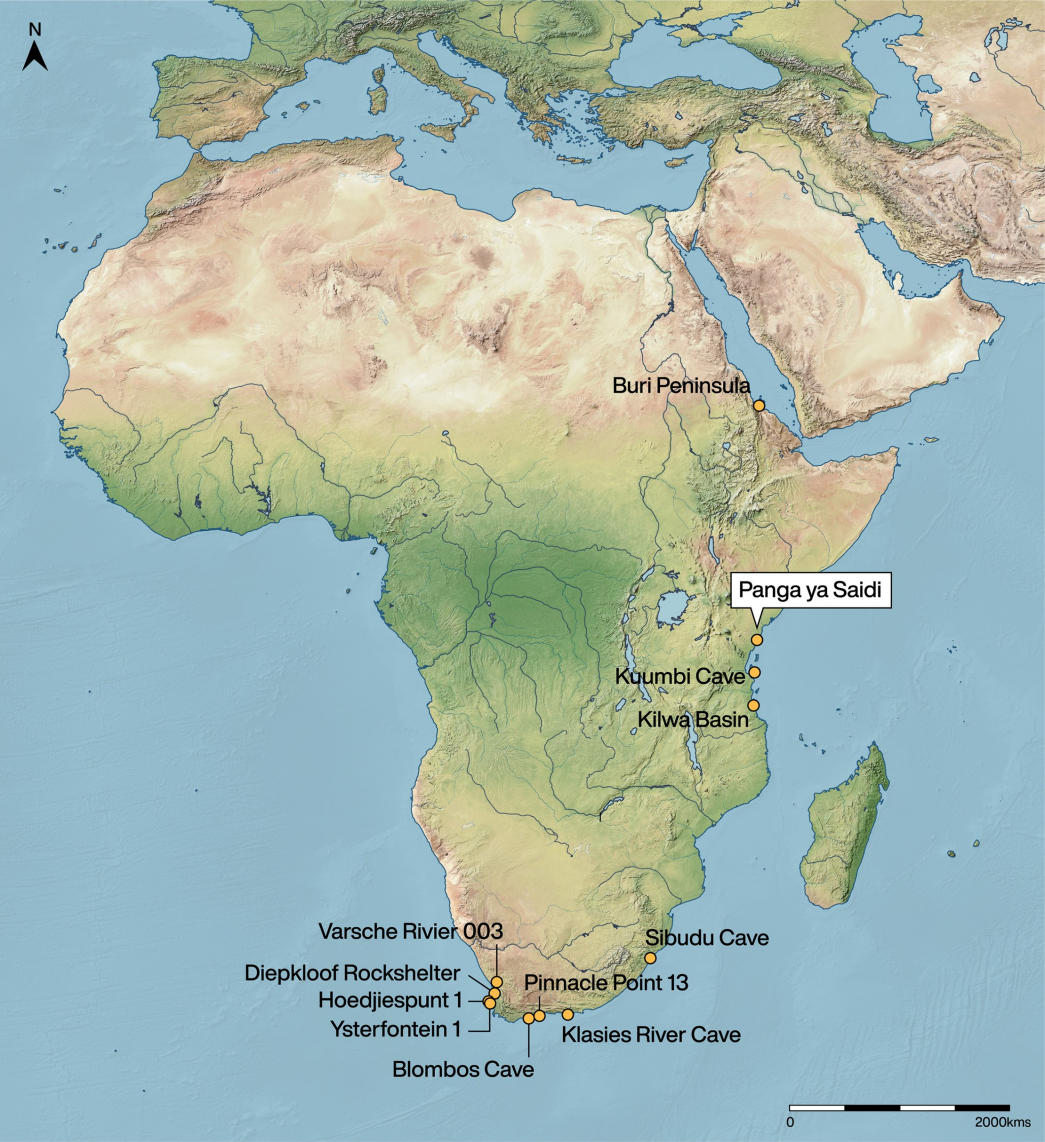
Pleistocene-aged sites with marine resource evidence as mentioned in text. The map was created using QGIS 3.12 (https://qgis.org/en/site/) and uses Natural Earth vector map data (https://www.naturalearthdata.com/downloads/).

In South Africa (Fig 1), direct evidence for coastal engagement appears before 150 kya, disappears by 50 kya, and re-emerges with increased intensity at the Holocene-Pleistocene transition [9, 18]. The oldest South African evidence can be found 164 kya (MIS 6) at Pinnacle Point (PP13B), where throughout the MSA people collected up to 25 marine invertebrate categories (identified to various taxonomic levels) for food and aesthetic purposes [19, 20]. Low-intensity marine engagement continues at Pinnacle Point and Hoedjiespunt 1 during MIS 5e [21, 22], and by 100 kya there is evidence for coastal habitat use at sites like Blombos Cave, Klasies River, and Ysterfontein 1 (e.g., [23–25]). Analysis of mollusc remains show that people at these sites considered foraging cost, transport, and perishability of resources, suggesting an early trajectory towards systematic and planned marine engagement [24, 26, 27]. Towards the end of the MSA, inland sites in South Africa such as Sibudu Cave [15] and Diepkloof Rock Shelter [16], as noted above, begin to show evidence of marine resources, though the nature of the relationship with the coast is unclear [9]. However, the abundance of South African data, including those from inland sites, indicate that the acquisition and transportation of coastal materials was not random or opportunistic, and suggests planned movement of people and resources that has implications for understanding changes in subsistence, mobility, and broader settlement structure [11].

Eastern Africa has comparatively less evidence for Pleistocene coastal engagement, possibly due to a research focus on the more recent Swahili trade era. Local maritime adapted societies were present from at least the 3^rd^ century along the coast of Africa, with the coastal lifestyle well-established by the Early Iron Age [28]. On Zanzibar (Unguja) Island, Kuumbi Cave provides stratified evidence for long-term coastal engagements beginning c.18 kya [29–31], although these trends are complicated by processes of sea level rise and island formation during the terminal Pleistocene and early Holocene. In the Kilwa Basin of Tanzania, Beyin and Ryano [32] reported on two surface scatter localities (Mnaraeka and Mapimbi) 4–5 km from the modern coast. These sites are both undated, but have medium-sized Levallois and blade cores, suggesting MSA occupation on the coastal landscape. They also report test excavations at two nearby sites in the Kilwa Basin, however pottery was found throughout suggesting a more recent age [32]. In Eritrea, Beyin and Shea [33] identified 17 coastal and peri-coastal open-air sites around the Buri Peninsula. These undated scatters were mostly classified as LSA based on lithic typologies, though some were associated with MSA tools, but it is unknown whether a sub-surface component exists. Finally, a 6.5 km stretch of exposed reef terrace (Abdur Reef) with embedded MSA tools was identified along the Buri Peninsula in Eritrea [34]. This reef was proposed to be 125 kya old, though the complex formation processes and high potential for redeposition of artefacts may render this age unreliable [35], however this is near to where Beyin and Shea [33] identified surface scatters with LSA and MSA tools. These examples all hint that the eastern African coastline was occupied at least from MIS 5 into the Holocene, but offer little in the way of a regional comparison with South African data. These limited reports of stratified or well-dated sites preclude a wider understanding of marine engagement and resource use in this region.

In this study, we report a 67,000 year long marine mollusc sequence at Panga ya Saidi (PYS), a near-coastal cave site in Kenya. PYS contains the oldest stratified evidence of coastal resources in eastern Africa, and provides the first glimpse into the timing, nature and trajectory of marine resource engagement in this region. PYS is also the first stratified site in Africa with an archaeological record from MIS 5–1, possibly due to the peri-coastal setting, and we use these data to provide the continent’s first long-term socio-economic sequence of coastal engagement. Sites located directly on the modern coast, particularly those in South Africa, have large gaps in their chronological sequence. Crucially, the period from 50–14 kya tends to be absent, which appears to coincide with an increase in marine adaptations. The MIS 4–2 global reduction in sea levels pushed the South African coastline an additional 10–15 km away, possibly out of the daily range of Pleistocene forager groups [7]. Pleistocene sites located inland (beyond 10 km from the coast) with evidence of marine resources are rare, suggesting that proximity to the sea was an important factor. However, if people moved to be closer to the coast during MIS 4–2 reduction in sea level, then the LSA evidence of their marine exploitation would become submerged on the continental shelf as sea levels subsequently returned to modern levels [7, 32, 36]. Or, if some coastal caves continued to be used in the LSA, those archaeological deposits may have since been flushed out by the periodic advance and retreat of the sea [7, 9, 23]. PYS is far enough from the coast to be protected from these effects, while the steep coastal shelf kept marine resources at a consistent distance until the early Holocene.

Precursors of marine engagement, or occasional contact with coastal resources, may be difficult to identify archaeologically, such as newly exploited habitats or changes in the uses and value of marine resources. Material culture associated with marine engagement (e.g., hooks, nets, baskets) are typically made from perishable material, and are often invisible in the archaeological record. The mollusc record is often comparatively better preserved and more abundant, providing the best proxy evidence by which to trace engagement with marine habitats and the resources contained within them. When deposits are situated away from the coast, mollusc data also provide critical insights into logistical planning, residential mobility, and/or trade networks, although this may be complicated by differential processing and transportation of the meat and/or shell [7, 26]. Finally, molluscs have the potential to inform multiple dimensions of the human experience, as they can represent subsistence activities, symbolic ornaments, or both. In order to understand the relationship to coastal resources at PYS, we must examine all mollusc data, focusing on characterising the major transitions through time. As this is the first site of its type in eastern Africa, our focus is on the site-specific implications. While we make no claims for the regional extrapolation of these data, we identify several trends that should be investigated using comparable regional data in the future. Ultimately, this study is a first look at the ways in which marine and freshwater molluscs increase our understanding of subsistence and symbolic behaviours, and long-term, broader-scale behavioural trends in eastern Africa.

## Site location and description

**Fig 2:**
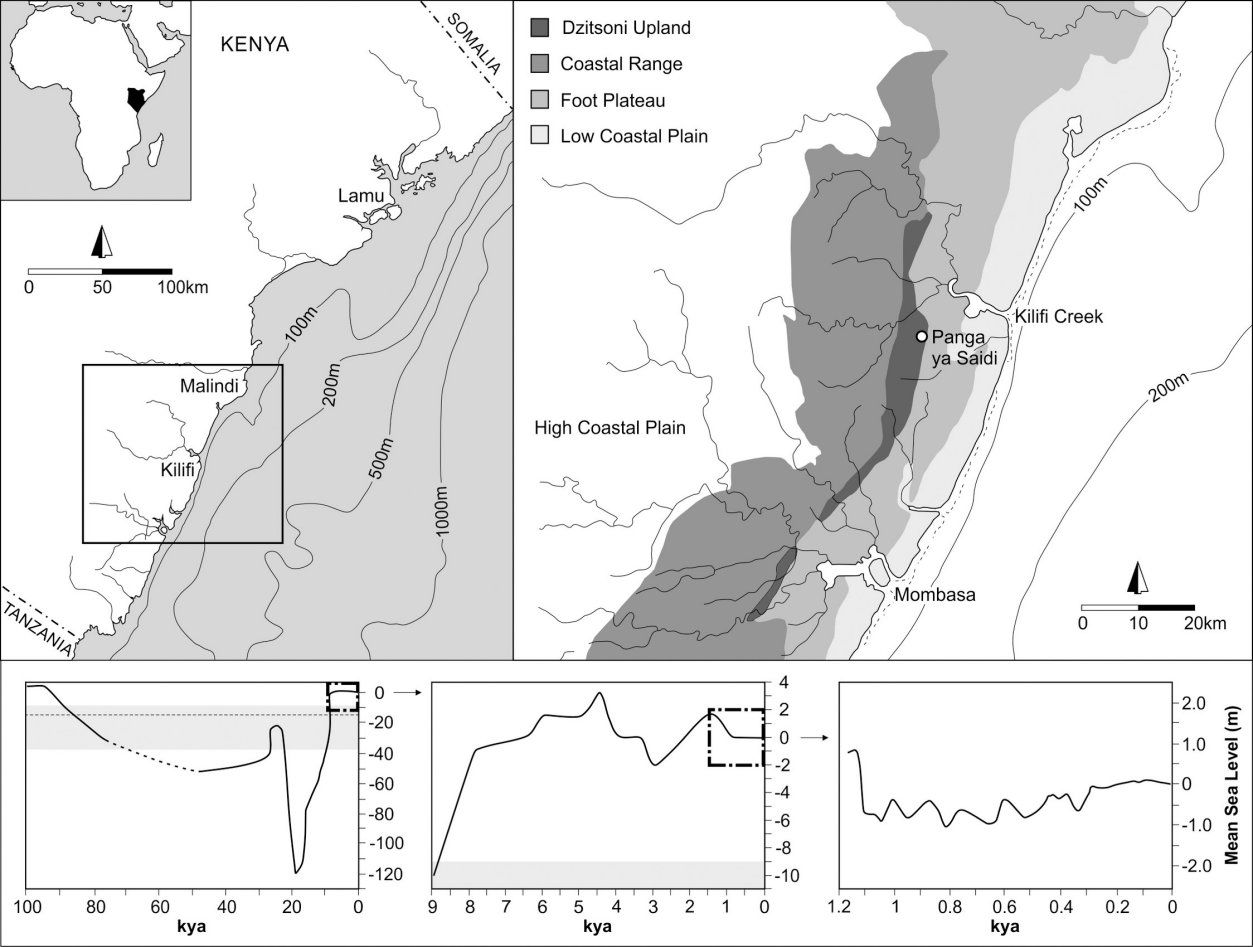
Southern Kenyan coast showing major physiographic divisions, bathymetric data, and mean sea level (MSL). Dashed line in lower left sea level curve at -15 m below MSL represents mean depth of the Kilifi Creek channel. Grey shading in lower left and centre sea level curves indicates the maximum depth at the channel mouth (-38 m) and maximum channel depth at the entrance to Kilifi Creek Lagoon (-9 m).

PYS is situated in the Kilifi District, at c.150 m above sea level (asl) on the east facing escarpment of the Dzitsoni Uplands of southern Kenya. These uplands separate the coastal plains from the arid Nyika Plateau interior, which is dominated by a dry Acacia Thorn-Bushland. Positioned within the Zanzibar-Inhambane coastal forest mosaic (lowland moist and dry forest remnants), PYS lies c.8-9 km inland from the Kilifi Creek Lagoon (as the shortest straight-line distance to the current landward edge), and ≤15 km from the present coastline (Fig 2).

A range of local paleoenvironmental proxies, including stable isotopes on mammal teeth, tetrapod, and molluscan fauna, phytoliths, sedimentology, and magnetic susceptibility [37, 38], indicate a degree of continuity in tropical forest/woodland and grasslands through time. There was generally low variability in environmental conditions, with transitions between open/closed woodland environments. A shift to drier conditions after 78–73 kya, with increased grassland cover between 50 and 20 kya, returning to forest from the terminal Pleistocene [37]. In general terms, these records agree with broader East African data that highlight low amplitude environmental shifts across much of the MIS 4 to MIS 1, this particularly being the case nearer to the coast. Rainfall models for the Last Interglacial and the Last Glacial Maximum (LGM) indicate that the Nyika Plateau has been drier than the Nyali coast, largely due to marine influences buffering fluctuations in climate and environmental conditions [38].

The Kenyan coastal belt as it is currently configured results from a combination of eustatic sea level fluctuations, tectonic movement, isostatic adjustment, progradation and sedimentation [39–42]. Sea level fluctuations (Fig 2) are evidenced by marine coastal terraces dating to the late Pleistocene and Holocene, with raised reef complexes indicative of a sea level highstand dating to MIS 5 (c.6-7 m above present sea level) [41, 43]. On the western Indian Ocean sea levels dropped to c.110-115 m below present levels about 18–17 kya [41, 44]. This was followed by a gradual increase, interspersed by stillstand periods, to c.9 kya with sea levels reaching c.22 m below present levels. The mid to late Holocene is marked by sea level oscillations, with evidence coming from South Africa and Mozambique for fluctuations between 3.5 m above and 2 m below present sea level [41]. On the Kenyan coast, a mid-Holocene sea level highstand of c.4-6 m above present levels has also been reported [41]. Beach or dune ridge formation on the coast in the Kilifi District continued following post-glacial sea level rise, with radiocarbon ages obtained from shell samples from one ridge south of Kilifi falling within the last 1000 years [45], corresponding with the most recent drop in sea levels. PYS would have maintained its proximity to the coast of <20 km throughout the known period of occupation of the site, even with significant changes in sea level, due to the coastal shelf dropping c.125 m within 4–5 km of the modern coastline (Fig 2) [38, 46].

The expansive Kilifi Creek Lagoon (also known as Bandari ya Wari) is important to consider in this context. The interior of Kilifi Lagoon, with an estimated area of c. 6 km^2^, is considerably wider than the narrow channel mouth of the creek (at 4 km from the open lagoon), where the continuity of the coral reef is presently broken due to outflow of freshwater [42, 45, 47]. The western side of the lagoon is extensively covered with mangroves over an area of c. 3.6 km^2^, including two main tidal creeks, with significant mangrove diversity recorded across this area [47]. Representing a drowned river valley following postglacial sea level rise, this area would have been subsequently modified via sedimentation from fluvial and marine sources, forming tidal channels, mangrove swamps, tidal flats, and estuaries [48]. At 4 km in length and c. 500 m wide, the mean depth of the Kilifi Creek channel is 12–15 m below mean sea level (MSL), ranging from 35–38 m at the mouth, gradually increasing over the length of the channel to c.8-9 m where it enters the lagoon [49–51]. The Kilifi Creek Lagoon basin itself is comparatively shallow, with a mean depth of 5–7 m, and depths of several sampling points away from the channel ranging from c. 1.6–3.8 m [50]. The steep topography around Kilifi Creek, rising 10 m within 1–2 km of the shoreline, not only influences the morphology of the Kilifi Creek Channel, but also constrains the maximum extent of the lagoon by creating a spatially restricted tidal zone [52]. Based on the available sea level data (Fig 1) and the gradual decrease in depth from c. 38 to 9 m along the 4 km length of the Kilifi Creek channel from the mouth to the lagoon entrance, it is likely that sustained marine incursion through the channel and into Kilifi Creek Lagoon itself occurred after approximately 10 to 9 kya. The absence of geomorphological research within Kilifi Creek Lagoon, however, means that the impact of Holocene sea level oscillations and variability in sedimentary input on habitat formation and resource availability until at least c.1.1 kya are unable to be fully assessed.

**Fig 3:**
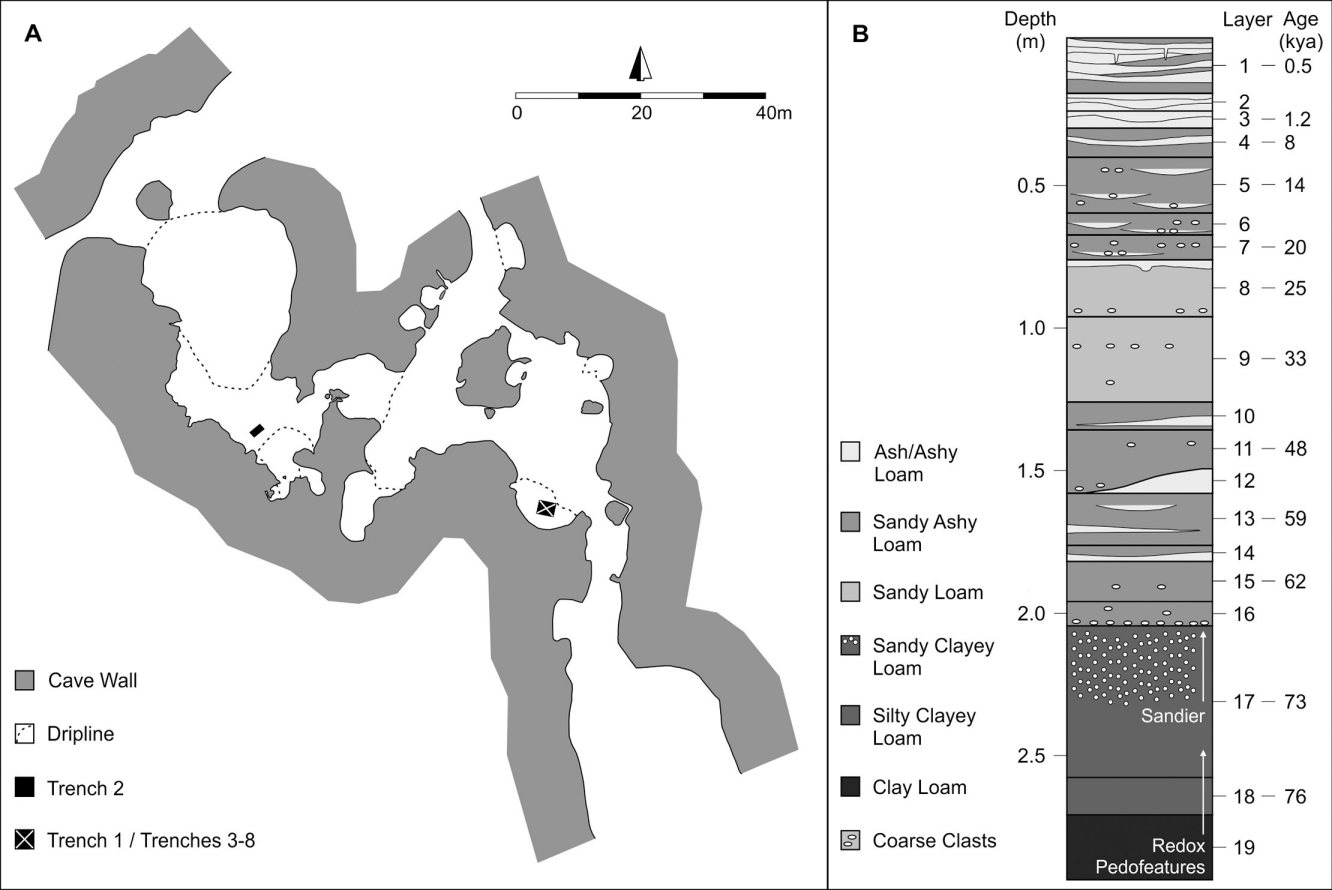
Panga ya Saidi site plan (A) and representative stratigraphic and chronological sequence (B).

PYS itself is a large limestone cave complex, comprising three main connected and partially roofed chambers [38]. Excavation has focused in the largest of the open chambers, with the main excavation block (Trenches 1, and 3–8) located in a sheltered alcove near the modern entrance (Fig 3A). The stratigraphic and chronological sequence is presented in detail elsewhere [37, 38, 53–57], with PYS preserving evidence for intermittent pulses of human activity 78–0.4 kya (Fig 3B). Lithic refits, bead typologies, and the presence of visible horizons support minimal movement of finds between stratigraphic layers [53, 58]. Here, we focus on the broader shifts in mollusc discard patterns, rather than the age of individual finds.

With the exception of symbolic materials [53], evidence of the transportation and/or consumption of marine and freshwater resources at PYS is limited in comparison with terrestrial taxa [37, 56]. Based on analyses of tetrapod assemblages recovered from Trenches 3 and 4 (total NISP = 5239; total MNI = 358), the Cephalophini and Neotragini (as bush adapted, small browsing bovids) dominate the PYS sequence, particularly in Layers 19–17 (MIS 5) and Layers 3–1. There is increasing diversity in the vertebrate fauna represented from Layers 16–13 (MIS 4) through to Layer 4 (Middle Holocene), with an increase in grazing bovids (e.g., Alcelaphini, Bovini, Reduncini) relative to browsers. This shift is particularly apparent in Layers 12–9 (MIS 3) and Layer 8 (LGM). Suids (e.g., warthog) follow a similar trend to the bovids, being represented throughout the PYS sequence from Layer 16 (MIS 4). These data suggest that while environmental and habitat shifts are evident (as noted above), there was always a mixture of closed forest and open woodland within the vicinity of the site, coupled with the sporadic appearance of open grasslands [37, 38]. In line with the zooarchaeological evidence from the South African sites dating to MIS 5 and MIS 4 [12], the vertebrate faunal evidence therefore highlights the terrestrial economic focus of the occupants at PYS.

## Materials and methods

All necessary permits were obtained for the described study, which complied with all relevant regulations. Excavation of this material was carried out under research licenses NCT/RR1/12/1/SS/541, NCST/RCD/14/013/944, NACOSTI/P/15/7427/5051, NACOSTI/P/16/7427/11341, granted by the Kenyan National Commission for Science, Technology & Innovation. Specimens reported in this study from Trenches 1–3 are housed at the National Museums of Kenya (Nairobi, Kenya), Trench 4 are temporarily housed in the University of Queensland Archaeology Laboratory (Brisbane, Australia), and Trenches 5–9 are temporarily housed in the Department of Archaeology, Max Planck Institute for the Science of Human History (Jena, Germany). Material studied in Kenya was undertaken via affiliation with the National Museums of Kenya’s (NMK) Archaeology Section in the Department of Earth Sciences. Material exported for analysis was permitted through a Material Transfer Agreement from the NMK. All material will be returned for permanent curation at the National Museums of Kenya in Nairobi.

All aquatic molluscan faunal remains recovered from PYS were identified to the lowest taxonomic level possible (e.g., family, genus, or species) with reference to published material [58–65] and comparative specimen collections of the Department of Archaeology at the University of Sydney. A previous study by d’Errico and colleagues [53] included analyses of symbolic aquatic mollusc shells excavated from 2010–2013 (Trenches 1, 3 and 4), while the present study analysed subsistence shells as well as symbolic molluscs excavated between 2010 and 2017. This comprised material from all excavation units (Trenches 1, 3–8). All identifications were checked using the World Register of Marine Species [66] to ensure consistency in the recorded nomenclature. Shell condition was noted for all samples, including degree of preservation, indications of burning and any observations on breakage patterns that might indicate processing or extraction of the meat. Any alteration by natural processes was also recorded, including beachrolling, boring and epibiont adhesions on the inner surfaces of the shell (following Szabó [67]). The identification of the symbolic/modified shell specimens is based on the information provided by d’Errico et al. [53]. As further assessment of the PYS disc/circular shell beads (*n* = 41) is required to determine whether they have been manufactured from the shell of marine or terrestrial taxa, these specimens have been excluded from the following analyses.

The Number of Identifiable Specimens (NISP) and Minimum Number of Individuals (MNI) were recorded for each taxonomic category. MNI counts were based on the preservation of non-repetitive elements determined on a taxon-specific basis (following Harris et al. [68]). For bivalves, these features were greater than 50% of the left and right hinge/umbo, for gastropods these included greater than 50% of the spire/apex, aperture, posterior or anterior canal, or the columella. To avoid the effects of aggregation with MNI [69], these values have been calculated by Layer (as discrete depositional units within occupational phases) rather than individual excavation contexts. Most analyses undertaken within archaeomalacology use MNI as the primary measure of abundance (e.g., [29, 70, 71]), however, due to assemblage size limitations, differential levels of fragmentation across taxa and the incorporation of modified shell specimens, all analyses are based on NISP to ensure comparability of data. Plotting NISP:MNI by Layer (S1 Fig), combined with results of Pearson correlation for the total assemblage (*r* [12] = 0.97, *p* < .001, *r*^2^ = 0.95), the Ostreidae (*r* [9] = 0.99, *p* < .001, *r*^2^ = 0.99), Potamididae (*r* [7] = 0.98, *p* < .001, *r*^2^ = 0.97) and Iridinidae (*r* [10] = 0.77, *p* = .009, *r*^2^ = 0.60), indicates a significant positive relationship between these measures of abundance. These results highlight the suitability of NISP as a measure of taxonomic abundance for further analysis of the PYS molluscan data. These data have also been volume corrected according to the total litres excavated per Layer, thereby providing a comparative measure of shell density, with these values presented as NISP/m^3^ (NISP/[litres x 0.001]).

Comparative analyses have frequently used marine shell density as a proxy measure of exploitation and/or transport intensity, often expressed as kg/m^3^ or MNI/m^3^ (e.g., [1, 12, 72, 73]). These authors acknowledge issues with these data, most notably that they are sparse and often inconsistent between sites, creating difficulties in using density values as a measure of the intensity of resource use. Jerardino [72] has also convincingly argued against using shell density as a direct comparative measure within and between sites, highlighting the range of complicating factors including sea level fluctuations, geological and geomorphic context, assemblage composition, and taphonomic processes, combined with distance to coast and shell transport decisions. Here we evaluate shell density as a basis to establish the relative shifts and directional trends through the PYS sequence. Rather than assessing rates of accumulation (e.g., [74, 75]), this approach provides a comparative measure of geometric density (sensu Bailey and Craighead [76]) that accounts for significant variation in layer characteristics and volume (as established by Shipton et al. [31]). As noted by Jerardino [72], shell densities are often inversely correlated with distance to source, therefore the volume corrected data are considered relative to taxonomic, ecological, and available geomorphic information [77].

Assessment of assemblage richness is based on the number of taxa (NTAXA) recorded per layer. To calculate NTAXA, the original taxonomic categories were grouped to the highest common level (e.g., family, genus) where appropriate, in addition to excluding two family level categories (Cypraeidae and Potamididae) and the Indeterminate Shell category. This conservative approach ensures that all taxa are mutually exclusive, removing the potential for multiple counting of individuals across categories [78]. Assemblage representativeness is assessed via a species area curve and nestedness analysis (following Lyman [78]; Wolverton et al. [79]). Plotting NTAXA with sample size (NISP) provides an indication of sampling to redundancy, whereby new taxa are not included with any increase in sample size [78, 80]. Visual inspection of the PYS species area curve (S2 Fig) indicates sampling to redundancy for the molluscan assemblage. As faunal assemblages with low richness should nest compositionally with samples exhibiting higher richness when drawn from the same community, a nestedness analysis will indicate whether samples with differing NTAXA are subsets. Nestedness temperature values provide a quantitative measure of this relationship, ranging between 100° (no nestedness) and 0° (perfectly nested) [78, 81]. The nestedness matrix and temperature value of 15.7^o^ (S1 Table) indicates a high degree of nestedness across the PYS samples. Together these measures indicate representativeness and adequacy of sample size for the following analyses.

## Results

### PYS mollusc assemblage characteristics

**Fig 4:**
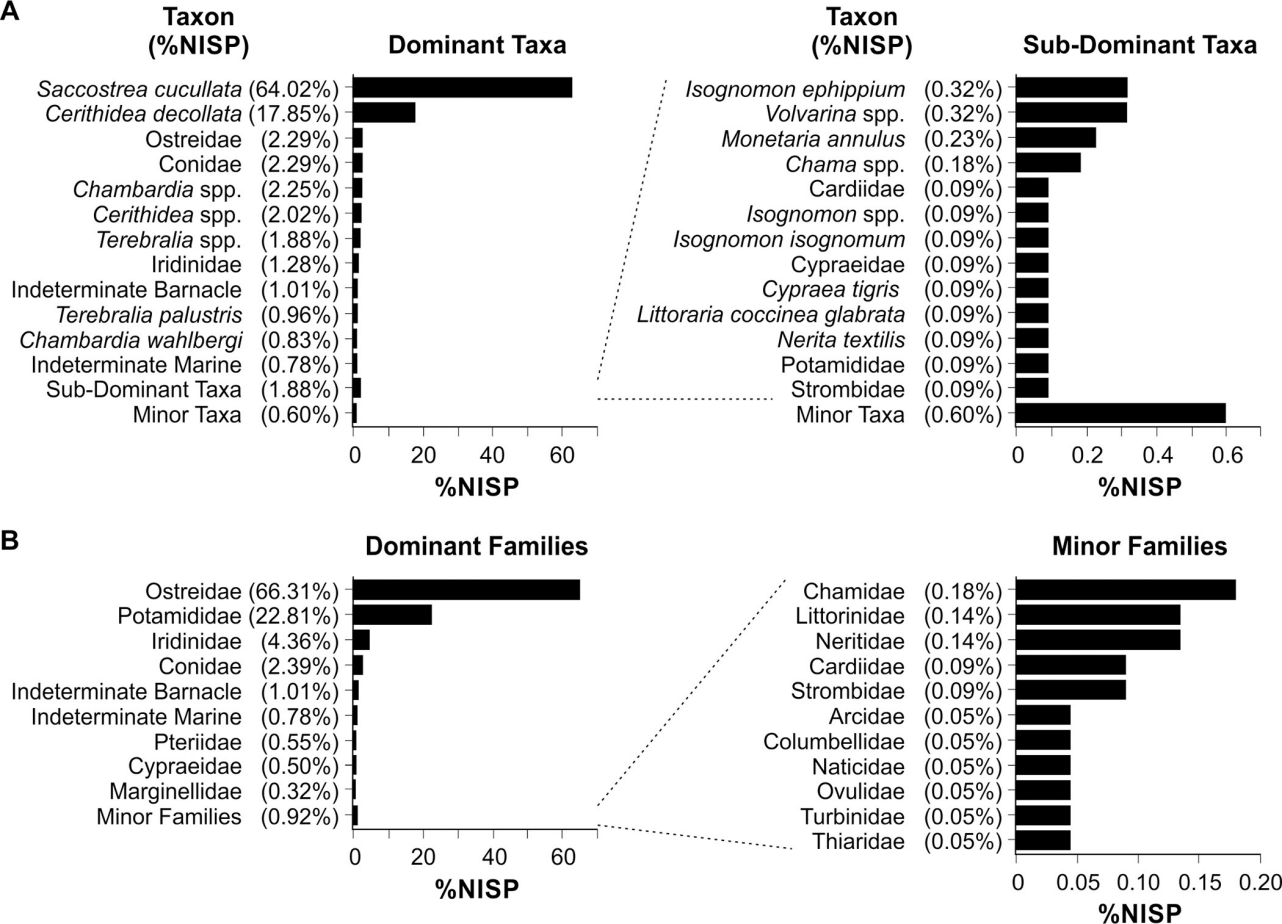
Proportional contribution of taxa to the total PYS molluscan assemblage (A) and the proportional contribution of taxa at family level (B).

**Fig 5:**
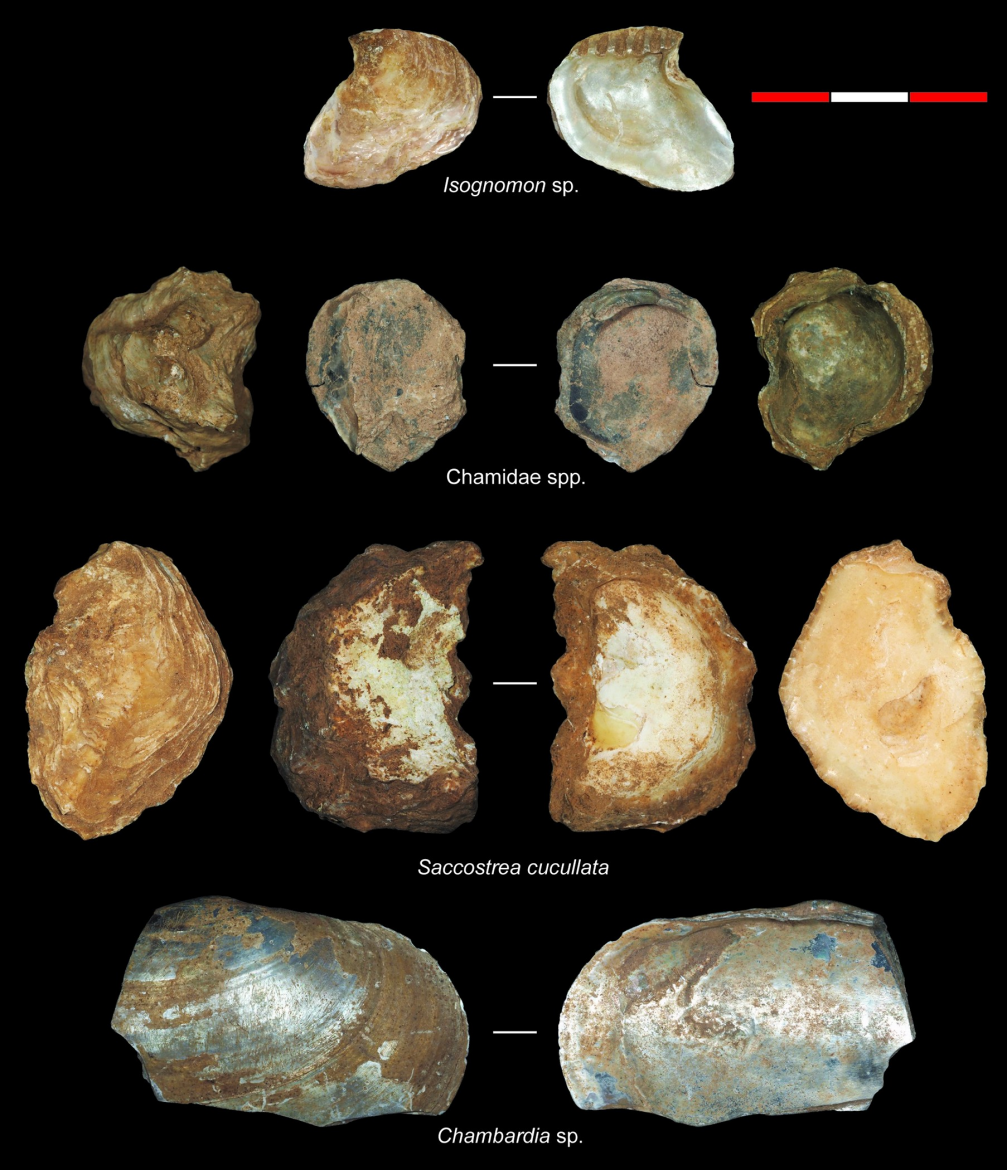
Examples of the marine and freshwater bivalve taxa recovered from PYS.

**Fig 6:**
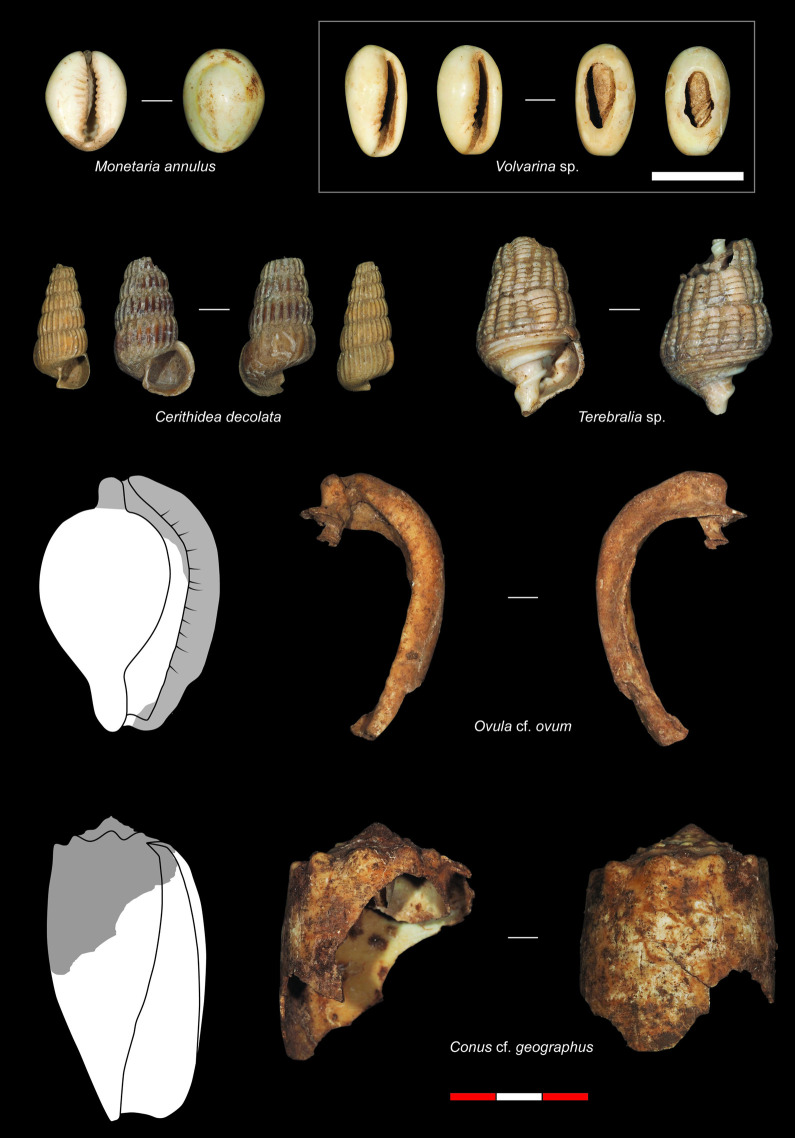
Examples of the subsistence and symbolic marine gastropod taxa recovered from PYS.

The total molluscan assemblage (2179 NISP) contains 38 invertebrate taxonomic categories (37 mollusc, 1 one crustacean), attributed to different taxonomic levels depending on the level of specimen preservation (Tables 1 and 2 presenting NISP and MNI respectively). There are 23 marine gastropods (579 NISP), one freshwater gastropod (1 NISP), nine marine bivalves (1464 NISP), three freshwater bivalves (95 NISP), one indeterminate shell (18 NISP) and one crustacea (22 NISP) category. This represents a relatively low degree of taxonomic richness, although as these data largely reflect marine invertebrates, low richness is likely linked to site location and distance to coast. The dominant taxon recovered from PYS is the hooded, mangrove or rock oyster *Saccostrea cucullata* (64.0% NISP), followed by the truncated mangrove snail *Cerithidea decollata* (17.9% NISP). All other taxa contribute less than 60 NISP per taxon to the total assemblage (≤ 2.7% NISP), with 13 taxa each contributing <0.05% NISP (or a single specimen per taxon) (Fig 4A). When viewed at family level (Fig 4B), the Ostreidae dominate, followed by the Potamididae gastropods (22.8% NISP) and the freshwater Iridinidae bivalves (4.4% NISP). The Conidae gastropods (cone shells, 2.4% NISP), Pteriidae (feather oysters, 0.6% NISP) and Cypraeidae (cowries, 0.5% NISP) make minor contributions to the total assemblage, with a further 11 families contributing a combined 0.9% NISP. Examples of the bivalve taxa recovered from PYS are illustrated in Fig 5, with examples of subsistence and symbolic gastropod specimens shown in Fig 6.

**Table 1:** PYS invertebrate NISP by Layer.

			Layer												
Class	Family	Taxon	1	2	3	4	5	6	8	9	10	11	15	16	Total
Bivalvia (M)	Arcidae	*Anadara* sp.	1												1
	Cardiidae	Cardiidae			1		1								2
	Chamidae	*Chama* spp.	1			1	2								4
	Ostreidae	Ostreidae	9	7	14	19			1						50
		*Saccostrea cucullata*	14	26	95	717	478	61				3	1		1395
	Pteriidae	*Isognomon* spp.		1				1							2
		*Isognomon ephippium*	1			2	4								7
		*Isognomon isognomum*					2								2
		*Pinctada* spp.					1								1
Bivalvia (F)	Iridinidae	Iridinidae	5		6	5	10		1		1				28
		*Chambardia* spp.	12	25	11							1			49
		*Chambardia wahlbergi*	5	6	2	1		3		1					18
Gastropoda (M)	Columbellidae	cf. *Pyrene* sp.		1											1
	Conidae	Conidae			1				3	15	29	1		1	50
		*Conus ebraeus*				1									1
		*Conus* cf. *geographus*											1		1
	Cypraeidae	Cypraeidae			1			1							2
		*Cypraea tigris*	1										1		2
		*Monetaria* sp.										1			1
		*Monetaria annulus*	1	2	1		1								5
		*Monetaria moneta*		1											1
	Littorinidae	cf. Littorinidae						1							1
		*Littoraria coccinea glabrata*					1					1			2
	Marginellidae	*Volvarina* spp.	4	1		1						1			7
	Naticidae	*Polinices* sp.	1												1
	Neritidae	*Nerita albicilla*	1												1
		*Nerita textilis*	2												2
	Ovulidae	*Ovula* cf. *ovum*										1			1
	Potamididae	Potamididae		1	1										2
		*Cerithidea* spp.	35	7	2										44
		*Cerithidea decollata*	179	150	34	8	10	2				6			389
		*Terebralia* spp.	12	6	5	9	9								41
		*Terebralia palustris*	2	4	10	4		1							21
	Strombidae	Strombidae			1					1					2
	Turbinidae	*Turbo* sp.							1						1
Gastropoda (F)	Thiaridae	*Thiara amarula*				1									1
Indeterminate	Indeterminate	Indeterminate Marine	1	2		7	6	1	1						18
Crustacea	Indeterminate	Indeterminate Barnacle		1	7	13	1								22
**Total**			**287**	**241**	**192**	**789**	**526**	**71**	**7**	**17**	**30**	**15**	**3**	**1**	**2179**
**Age BP (kya)**			**0.45**		**1.2**	**8**	**14.5**		**25**	**33**		**48.5**	**62**	**67**	
**Layer volume (m** ^**3**^ **)**			**0.94**	**0.91**	**1.28**	**1.35**	**1.52**	**1.6**	**1.38**	**2.03**	**2.3**	**2.06**	**0.5**	**0.79**	

Under Class (M) = marine, (F) = freshwater

**Table 2:** PYS invertebrate MNI by Layer.

			Layer												
Class	Family	Taxon	1	2	3	4	5	6	8	9	10	11	15	16	Total
Bivalvia (M)	Arcidae	*Anadara* sp.	1												1
	Cardiidae	Cardiidae			1		1								2
	Chamidae	*Chama* spp.	1			1	1								3
	Ostreidae	Ostreidae	2	1	2	1			1						7
		*Saccostrea cucullata*	6	8	27	272	140	18				3	1		475
	Pteriidae	*Isognomon* spp.		1				1							2
		*Isognomon ephippium*	1			1	2								4
		*Isognomon isognomum*					1								1
		*Pinctada* spp.					1								1
Bivalvia (F)	Iridinidae	Iridinidae	1		1	1	1		1		1				6
		*Chambardia* spp.	4	1	1							1			7
		*Chambardia wahlbergi*	5	2	1	1		2		1					12
Gastropoda (M)	Columbellidae	cf. *Pyrene* sp.		1											1
	Conidae	Conidae			1				3	15	29	1		1	50
		*Conus ebraeus*				1									1
		*Conus* cf. *geographus*											1		1
	Cypraeidae	Cypraeidae			1			1							2
		*Cypraea tigris*	1										1		2
		*Monetaria* sp.										1			1
		*Monetaria annulus*	1	3	1		1								6
		*Monetaria moneta*		1											1
	Littorinidae	cf. Littorinidae						1							1
		*Littoraria coccinea glabrata*					1					1			2
	Marginellidae	*Volvarina* spp.	4	1		1						1			7
	Naticidae	*Polinices* sp.	1												1
	Neritidae	*Nerita albicilla*	1												1
		*Nerita textilis*	2												2
	Ovulidae	*Ovula* cf. *ovum*										1			1
	Potamididae	Potamididae		1	1										2
		*Cerithidea* spp.	3	1	1										5
		*Cerithidea decollata*	111	108	12	2	5	1				2			241
		*Terebralia* spp.	2	1	1	3	2								9
		*Terebralia palustris*	1	2	4	2		1							10
	Strombidae	Strombidae			1					1					2
	Turbinidae	*Turbo* sp.							1						1
Gastropoda (F)	Thiaridae	*Thiara amarula*				1									1
Indeterminate	Indeterminate	Indeterminate Marine													
Crustacea	Indeterminate	Indeterminate Barnacle		1	1	3	1								6
**Total**			**148**	**133**	**57**	**290**	**157**	**25**	**6**	**17**	**30**	**11**	**3**	**1**	**878**
**Age BP (kya)**			**0.45**		**1.2**	**8**	**14.5**		**25**	**33**		**48.5**	**62**	**67**	
**Layer volume (m** ^**3**^ **)**			**0.94**	**0.91**	**1.28**	**1.35**	**1.52**	**1.6**	**1.38**	**2.03**	**2.3**	**2.06**	**0.5**	**0.79**	

Under Class (M) = marine, (F) = freshwater

**Fig 7:**
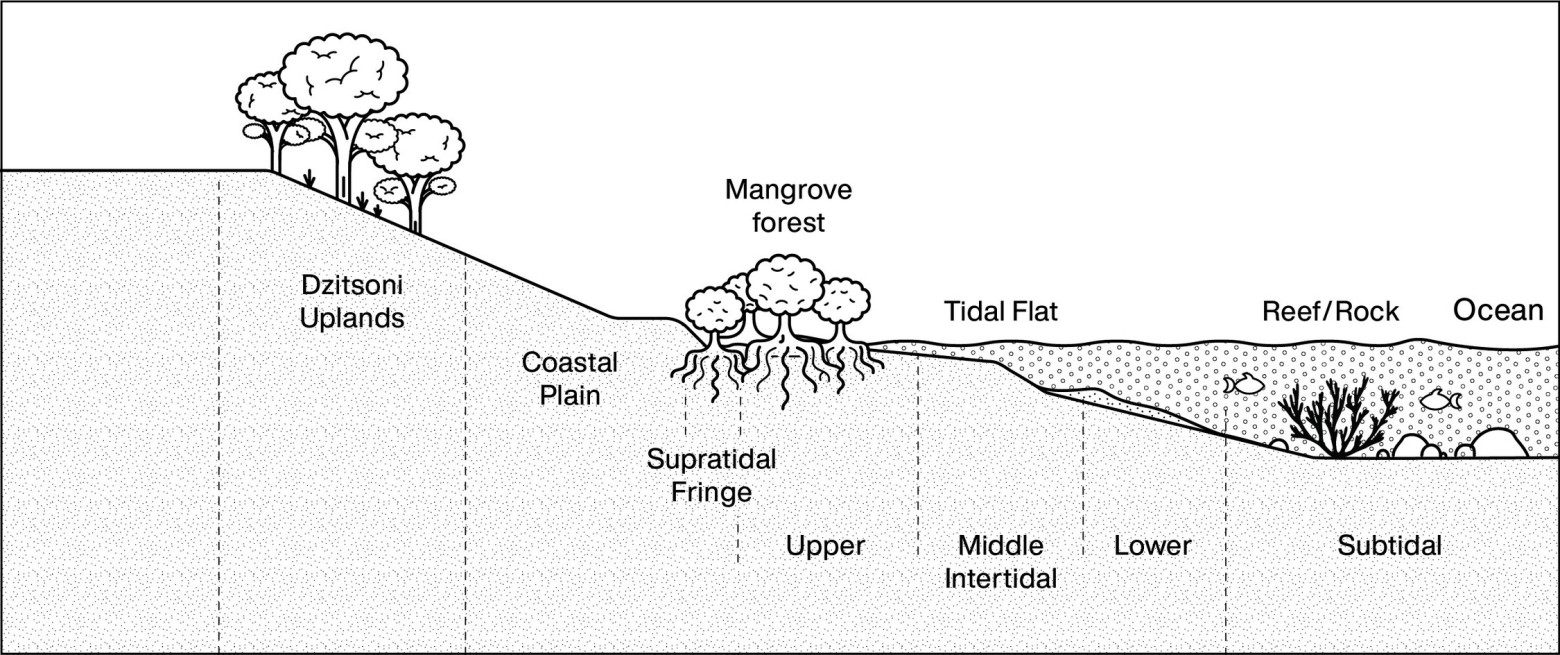
Schematic cross section of habitats on the Kenyan coast from the Dzitsoni Uplands to subtidal reefs.

The habitats represented by the PYS molluscan assemblage are dominated by intertidal/shallow subtidal rock/mangrove taxa (68.1% NISP) and supratidal mangrove taxa (20.2% NISP), the latter primarily climbing gastropods (see Fig 7 for schematic habitat distribution). Intertidal/subtidal reef/rock and supratidal/upper intertidal mangrove/mud species contribute 3.7 and 3.4% NISP respectively. Freshwater species (both still and running water) contribute 4.5% NISP combined, with intertidal and subtidal soft (sand/mud) taxa making a very minor contribution to the assemblage at 0.3% NISP.

### Comparative trends in mollusc discard

**Fig 8:**
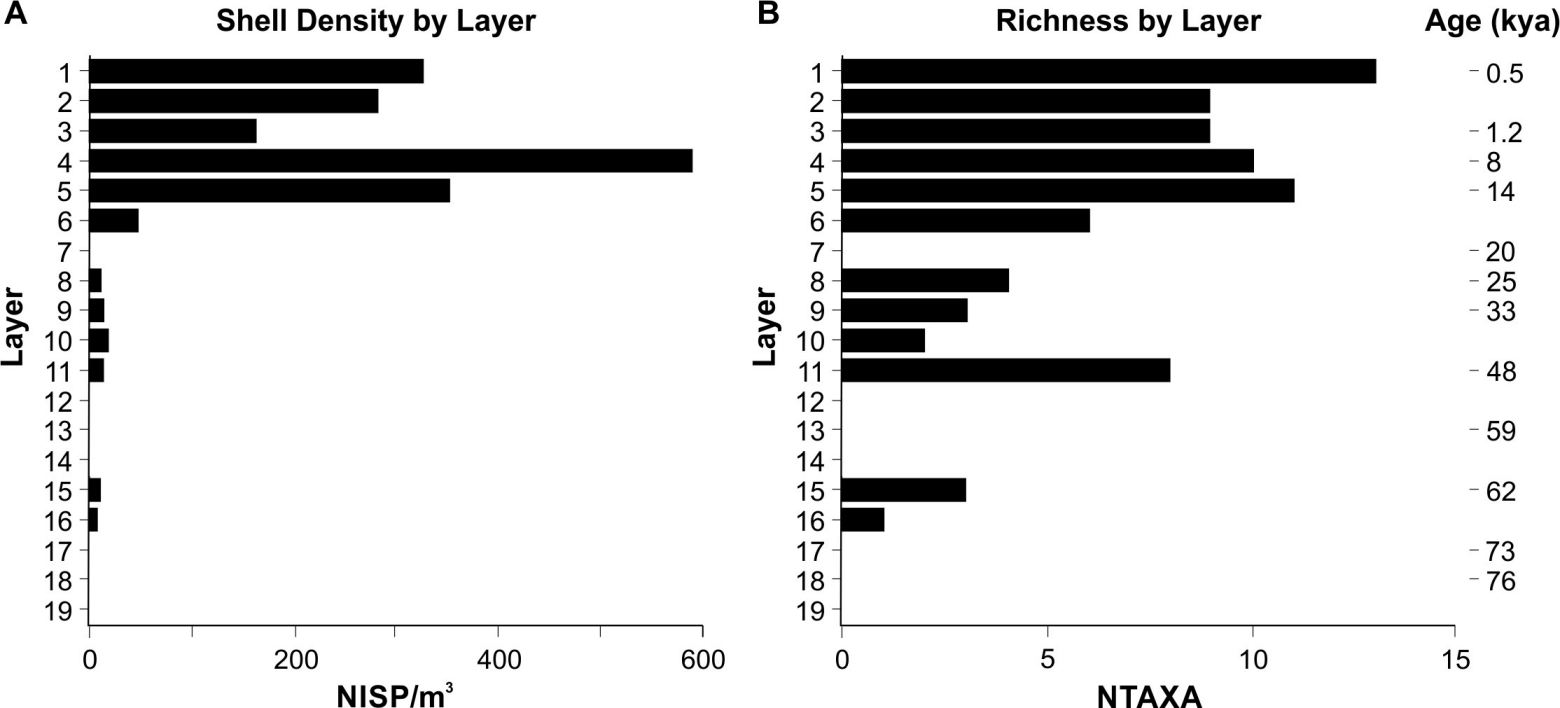
Shell distribution (NISP/m^3^) through the PYS sequence by Layer (A) and PYS assemblage richness (NTAXA) by Layer (B).

The distribution of aquatic shell (NISP/m^3^) by Layer through the PYS sequence (Fig 8A) shows minimal discard in Layers 16 and 15 (c. 67–62 kya, 1.3–6.1 NISP/m^3^) and Layers 11 to 8 (48–25 kya, 5.1–13 NISP/m^3^), with no shell recorded for Layers 7, 12–14 and 17–18. There is a relative increase in shell deposition from the terminal Pleistocene (Layers 6 to 1, 14–0.5 kya), although this exhibits a bimodal distribution, with shell density peaking in Layers 4 and 1 (585.5 and 319.8 NISP/m^3^, dating to 8 and 0.5 kya respectively) and a comparative decrease in Layer 3 (1 kya, 159.0 NISP/m^3^). Assemblage richness (Fig 8B) largely conforms to the trends seen in shell density, with lower NTAXA values earlier in the PYS sequence (apart from Layer 11), peaking in Layers 5 and 1. The higher richness value in Layer 11 reflects the first appearance of symbolic (*Monetaria* spp., *Volvarina* spp.) and economic (Littorinidae, *Cerithidea* spp.) taxa. Although represented by low NISP values, after Layer 11 these taxa only appear again in the PYS sequence variably between Layers 6 and 4. Although there appears to be a correlation between NISP/m^3^ and richness by Layer (S3 Fig), particularly during the Pleistocene where both sample size and NTAXA are low, this relationship becomes decoupled from the terminal Pleistocene to the late Holocene. This suggests that shifts in assemblage composition and discard reflects variations in human use and discard of marine and freshwater molluscs through time.

**Fig 9:**
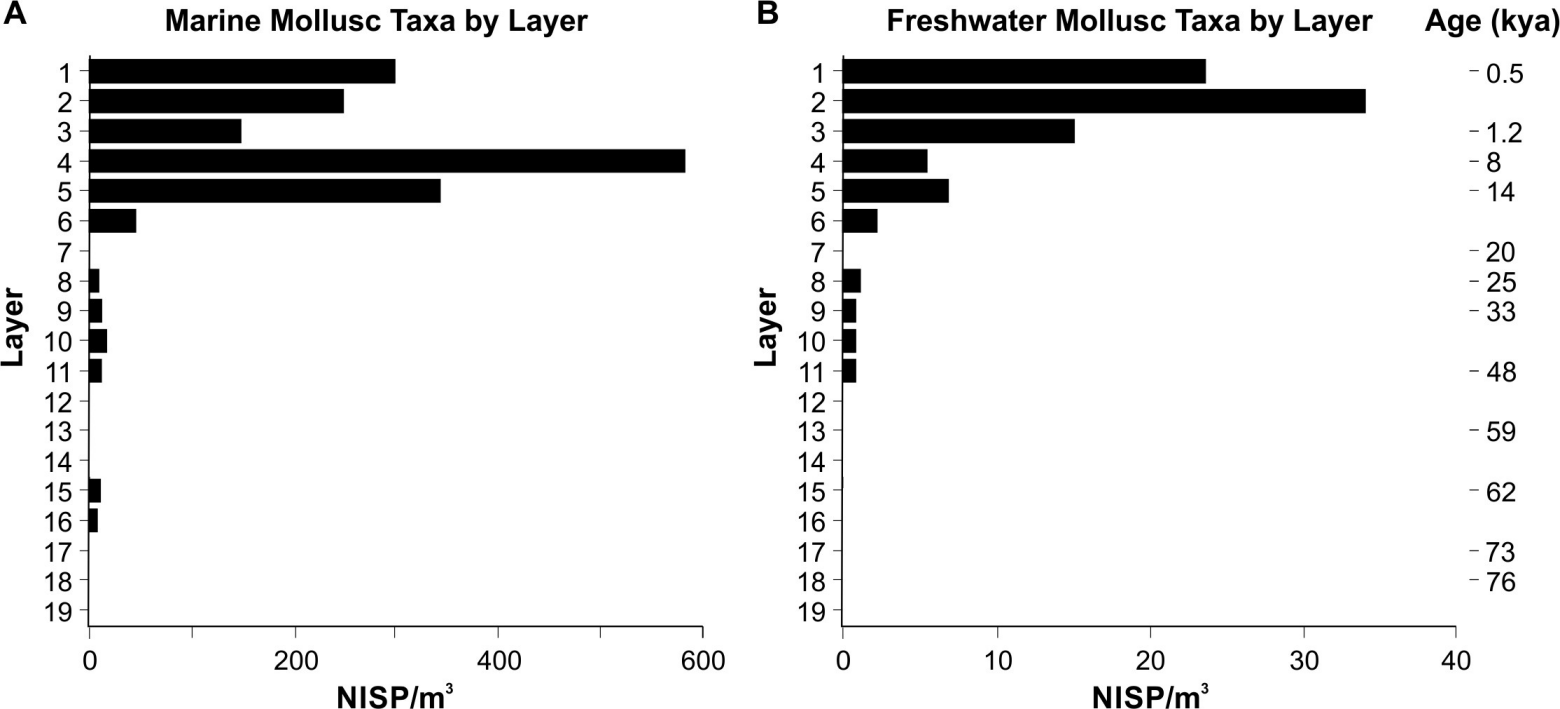
Distribution (NISP/m^3^) through the PYS sequence by Layer of marine (A) and freshwater (B) mollusc taxa. Note differences in horizontal scales.

The marine mollusc distribution (Fig 9A) replicates the trends seen in total aquatic shell distribution with increasing discard of marine taxa in the terminal Pleistocene (Layer 6, 42.6 NISP/m^3^). In contrast, the freshwater taxa (Fig 9B), which are predominantly bivalves within the Iridinidae family, indicate comparatively low densities between 48–25 kya (Layers 11–8, 0.4–0.7 NISP/m^3^), increasing from 14 kya (Layer 5, 6.6 NISP/m^3^) to peak between 1.2 and 0.5 kya (Layer 2, 34.0 NISP/m^3^). Breaking these data into the dominant taxonomic categories (at family or genus levels) reveals some important differences in the transport and discard of molluscan resources at PYS (S4 Fig). The Ostreidae are minimally represented until the terminal Pleistocene (0.7–1.5 NISP/m^3^, before 14 kya in Layer 6), peaking around 8 kya (Layer 4, 544.8 NISP/m^3^), following which they decline into the late Holocene. The mangrove gastropods *Cerithidea* spp. and *Terebralia* spp. (both within the Potamididae family) increase from Layer 5 (14 kya, 6.6 and 5.9 NISP/m^3^) to 0.5 kya (Layer 1, 228.1 and 14.9 NISP/m^3^ respectively). Although increasing, *Terebralia* spp. remain at a lower density (between 5.9 and 14.9 NISP/m^3^), whereas the *Cerithidea* spp. increase between 1.2 and 0.5 kya is considerable (from 28.2 to 228.1 NISP/m^3^). The Conidae and combined Cypraeidae and *Volvarina* spp. present different trends to those of the other dominant taxonomic categories. These specimens are identified as symbolic materials, either modified or unmodified (similar to the *Ovula* cf. *ovum* fragment in Layer 11, 48 kya), rather than being transported primarily for subsistence [53]. Absent in the late Holocene Layers, the Conidae peak sometime between c.48 and 33 kya (Layer 10, 12.6 NISP/m^3^), with single specimens present in Layer 15 (unmodified *Conus* cf. *geographus*; 62 kya) and Layer 16 (at c. 67 kya). The Cypraeidae/*Volvarina* spp. occur within Layers 15 and 11 (62 and 48 kya respectively) but increase to a minor degree from the terminal Pleistocene to peak in the late Holocene in Layer 1 (0.6–6.4 NISP/m^3^). These trends indicate differential transportation and discard of marine taxa to PYS from the coast through time for symbolic/aesthetic purposes.

**Fig 10:**
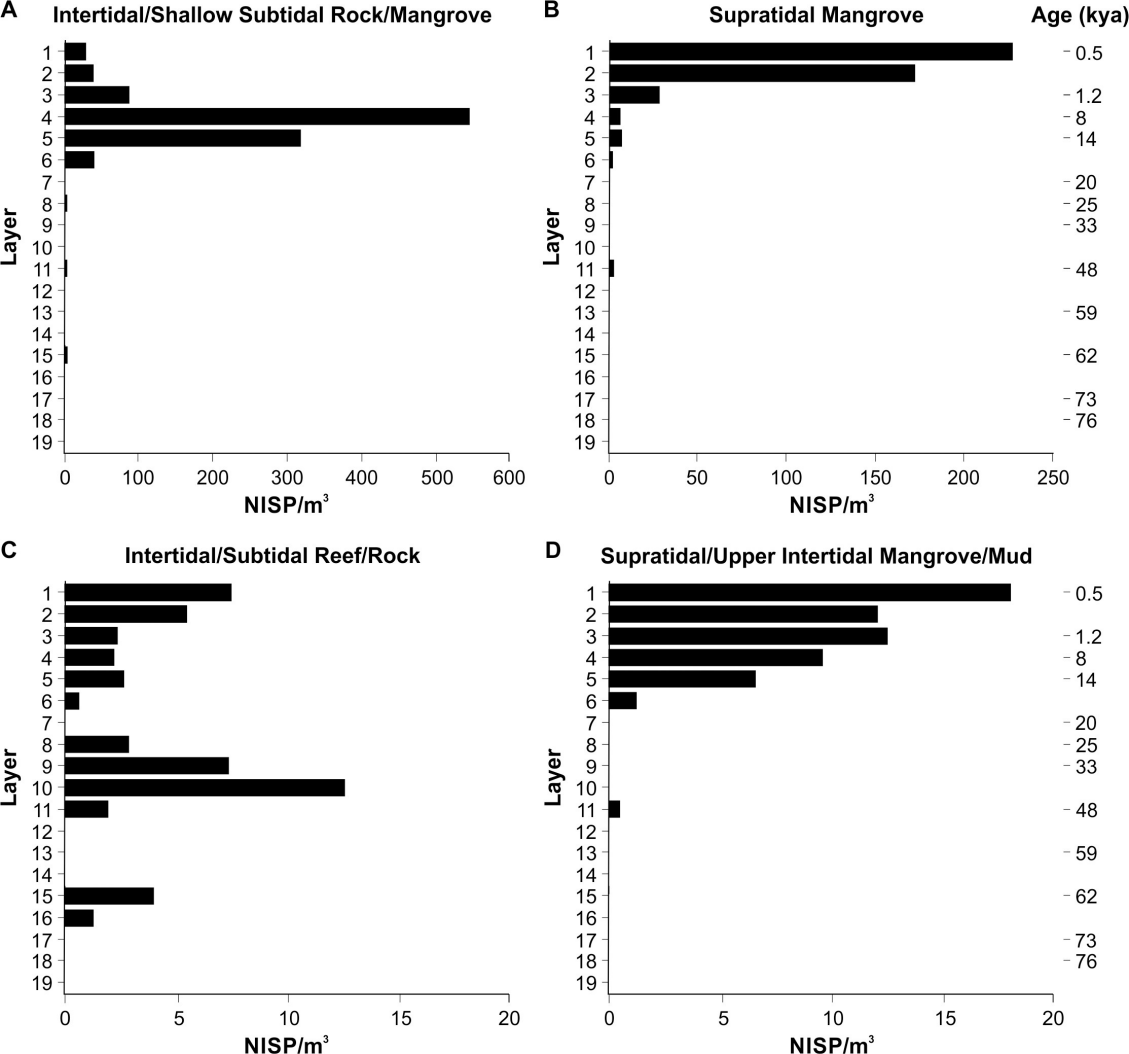
Distribution (NISP/m^3^) through the PYS sequence by Layer of taxa from broadly defined habitat zones. Intertidal/shallow subtidal rock/mangrove (A), supratidal mangrove (B), intertidal/subtidal reef/rock (C) and supratidal/upper intertidal mangrove mud (D). Note differences in horizontal scales.

These data also illustrate differential transport of taxa through time from a range of nearshore marine habitats. The density of taxa from intertidal/shallow subtidal rock and mangrove zones (Fig 10A) and supratidal/upper intertidal mangrove environments (Fig 10B and 10D) mirrors that seen for the Ostreidae and *Cerithidea* spp./*Terebralia* spp. (S4 Fig). This is unsurprising given the dominance of these taxa in the upper half of the deposit, highlighting a turnover in the assemblage from Layer 3 (1 kya), with a habitat shift from rock and/or mangrove reef to climbing mangrove taxa being transported inland. Although occurring at lower densities, taxa from intertidal/subtidal (reef and rock) hard substrate habitats (Fig 10C) present a multimodal distribution, peaking at 62 kya (4.0 NISP/m^3^), between 48 and 33 kya (12.6 NISP/m^3^), and in the late Holocene (7.5 NISP/m^3^).

**Fig 11:**
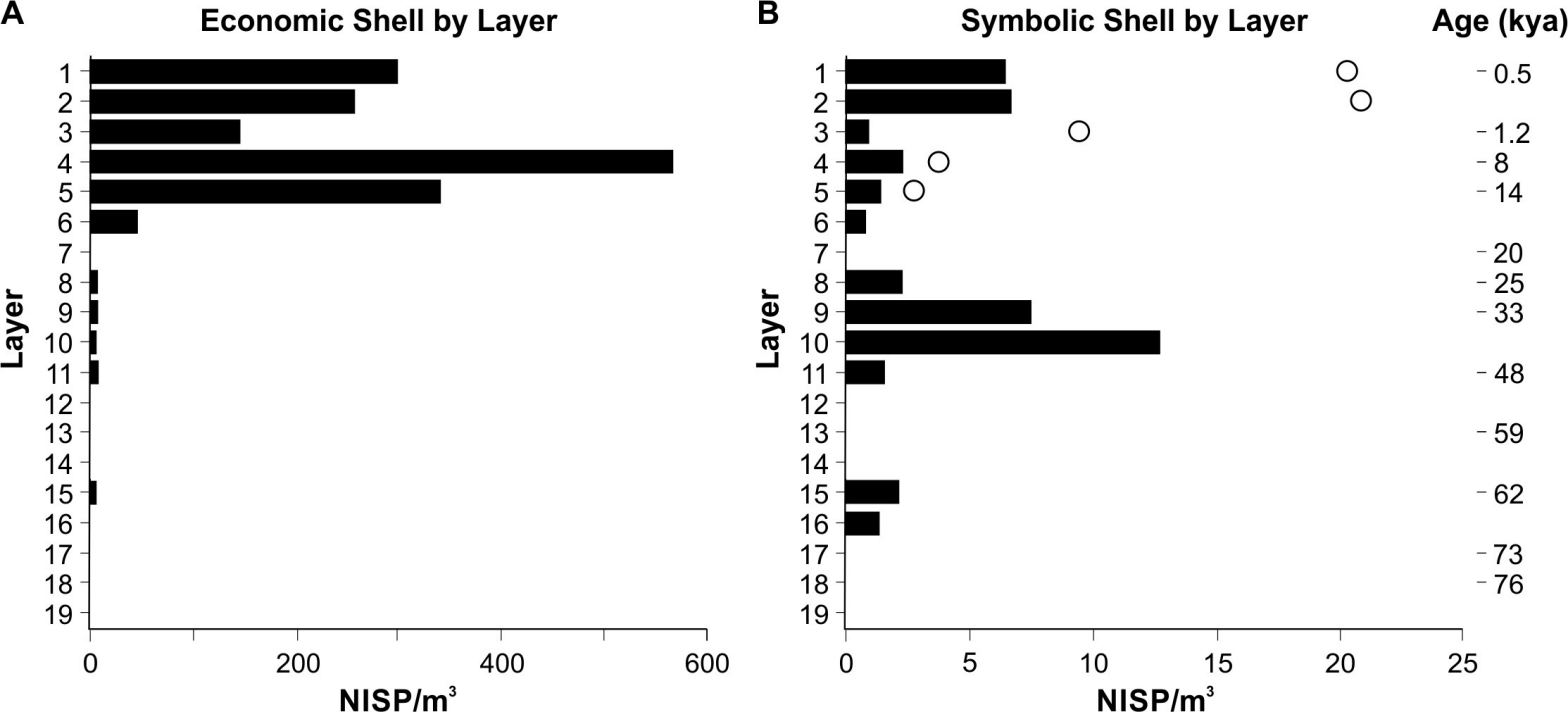
Distribution (NISP/m^3^) through the PYS sequence by Layer of economic (A) and symbolic (B) mollusc shell. White circles in (B) represent additional symbolic shell density including disc beads (as reported in d’Errico et al. [53]). Note differences in horizontal scales.

The distribution of economic (Fig 11A) and symbolic (Fig 11B) shell through the PYS sequence provides evidence of differential transport and discard of molluscs through time, and also supports the stratigraphic integrity of finds. Economic taxa are broadly defined to include those used for subsistence and/or symbolic or technological purposes (following Faulkner et al. [29]), the latter defined as edible taxa but where the shell may have been transported as raw material (without clear indications of modification on the shell). Following d’Errico et al. [53], the symbolic specimens include perforated gastropod shells and unmodified specimens more likely to have been transported for their symbolic/aesthetic appeal (e.g., Conidae, Cypraeidae) or as raw material (e.g., *Volvarina* spp.). There are clear pulses in discard of both economic and symbolic shell at PYS, with these occurring at different points through the sequence. There is very low density of economic taxa in Layer 15 (single *Saccostrea cucullata* specimen) and Layers 11–8 (e.g., *Saccostrea cucullata*, *Cerithidea decollata* and the Iridinidae, 0.4–5.8 NISP/m^3^). There is a major shift from Layer 6 (43.9 NISP/m^3^) with a bimodal distribution, where the major peaks occur in Layer 4 (8 kya, 567.0 NISP/m^3^) and Layer 1 (0.5 kya, 299.6 NISP/m^3^), with much higher levels of density/discard of shell across the terminal Pleistocene into the late Holocene. There are somewhat similar density values in symbolic shell material through the lower PYS Layers, although there is a major peak in Layer 9 (33 kya) and Layer 10 at much higher densities than the economic taxa (7.4–12.6 NISP/m^3^), similar to the intertidal/subtidal hard substrate distribution in Fig 10C). A decrease into Layer 6 (0.6 NISP/m^3^) is followed by a peak in the late Holocene Layers 2 and 1 (0.5 kya, 6.4–6.6 NISP/m^3^), albeit at lower densities than Layers 9 and 10. The inclusion of the circular beads (Fig 11B) also highlights the technological shift in symbolic materials noted by d’Errico et al. [53] for the Holocene, with higher densities through the lower portions of the deposit than seen previously.

## Discussion

The mollusc remains from PYS are the first genuine record of Pleistocene coastal engagement in eastern Africa, and the only long-term data available in Africa spanning the last 67,000 years. The site is terrestrially focused, as indicated by the available zooarchaeological evidence, isotopic data [37], and relatively consistent distance to the coast and access to coastal resources until marine incursion into Kilifi Creek in the early Holocene. As such, the PYS evidence does not directly speak to coastal resource use vs coastal adaptation, nor address models of coastal human dispersals out of Africa (e.g., [6, 82, 83]). What the PYS mollusc data does suggest, however, is that people were engaged with the coast by 67,000 years ago, and through time their relationship with marine environments shifted relative to resource distribution and density, concomitant with habitat reconfiguration. As an extension of this, given that the anthropogenic origin of these resources entails harvesting within marine environments and transportation at distance to an inland location, the evidence from PYS would also predict human use of landscape to some degree in coastal and near-coastal areas. It is apparent that throughout the period of human occupation at PYS, people were accessing molluscs for symbolic purposes and/or consumption, with pulses of shell discard occurring at approximately 67–62 kya, 48–25 kya, in the terminal Pleistocene, and the later Holocene. These data document an early symbolic engagement with coastal resources that shifted to include edible species being transported to the cave from the terminal Pleistocene through to the Swahili trade era. Based on current evidence, marine shell was not transported to PYS between 62 and 51 kya (Layers 14–12), the reasons for which are unclear. As molluscs were likely to have been an economic resource on the coast during this time, and while difficult to assess given the lack of comparative sites of the same antiquity between PYS and the coast, it is possible that the absence of shell may relate to field processing to remove the meat before transportation (as suggested by Bird et al. [84]; Cawthra et al. [77]; Dusseldorp and Langejans [26]; Jerardino [72]). Lithic materials continue to be discarded, as do terrestrial vertebrate fauna, although there is a marked transition in these taxa from initially proportionally even representation of browsers and grazers, followed by a significant increase in grazers in the latter part of this period [54].

The earliest mollusc data at PYS show a low level of engagement with coastal resources that is unlikely to be food related. Absent in Layers 19–17, the first evidence appears approximately 67–62 kya, in Layers 16–15, and accompanies a reduction in lithic flake size which indicates the transition to LSA technology [38, 54]. These oldest specimens are unmodified which supports their antiquity, while stratigraphically higher finds tend to be pierced for suspension. The shell in Layer 15 is attributed to *Conus* cf. *geographus* based on its retention of surface patterns. While small finds like disc beads may be susceptible to stratigraphic movement, this specimen is cup shaped and measures >5 cm long, suggesting that it would not be easily displaced in the stratigraphy. The *C*. cf. *geographus* is a carnivorous gastropod that kills small prey with venom that is powerful enough to be fatal to humans. While extremely dangerous to handle, the toxin is temperature sensitive and the flesh may be consumed after cooking [61, 85]. As there is no evidence for burning, it is likely then that the specimen was not collected live, but rather the shell was collected after it had washed up on shore from its shallow-reef habitat based on an appeal to aesthetic appreciation of the colorful patterned shell, or even a reverence for the dangerous snail. The smooth, symmetrical, and rounded *Ovula* cf. *ovum* specimen recovered from Layer 11 (48 kya) is also visually appealing and may be interpreted in a similar manner to the *C*. cf. *geographus*. These shells are isolated and bear minimal modification but were transported over 15 km from the coast to PYS suggesting they had some value. These are strong indicators for aesthetic appreciation, material engagement, and symbolic expression (sensu Jerardino and Marean [20]), and they are currently the only evidence for MIS 4–3 marine shell transport in eastern Africa.

Symbolic shell discard at PYS resumes in Layer 11 and peaks in Layer 10 (48 to 33 kya), with a grouping of Conidae spire beads. These rounded, perforated discs are the result of natural degradation on the beach, which erodes the shell apex to create an aperture [53, 86]. The grouping of these Conidae spires in Layer 10 indicates that there is minimal vertical displacement of these small finds. Although most do not bear human-made perforations [53], these molluscs were collected and transported to PYS in groupings (likely to be used as beads), and as such they demonstrate a new more systematic level of symbolic engagement with coastal resources. Previous research has shown that Layers 12–10 are associated with the highest proportion of large bovids in the faunal sequence [37], and the lithic density evidence suggests an increase in occupation intensity in Layers 12–11 and 7–8 [54]. These data combined with advances in social technology may suggest that population sizes and/or inter-group contact were increasing at this time, putting greater pressure on new modes of visual symbolism. The symbolic materials moved from the coast and deposited at PYS c. 67–25 kya may have been transported following direct acquisition, or via some form of exchange (e.g. [46, 57, 87]; see discussion in [88]). Although the coast is located at a distance beyond the generally accepted daily foraging range of 10 km (see below), and there is a lower density of economic/subsistence freshwater and marine molluscs prior to the terminal Pleistocene, the chert source for lithic manufacture is situated between PYS and the coast at a distance of approximately 7 km [54]. While this creates a level of uncertainty in the mechanism of movement, the increased density of aquatic molluscs from the terminal Pleistocene (Layers 6 to 1) that have the ability to stay fresh for several days implies a delay between collection and consumption. This may suggest that people ~67 kya were employing different strategies than those at ~25 kya, and could indicate a different level of planning, landscape use and/or mobility between c. 67–25 kya. This pattern should be explored further as more data becomes available from eastern Africa.

Economic molluscs are minimally represented in Layers 15 and 11–8 (average 2.7 NISP/m^3^) compared to the symbolic shell materials deposited across the same layers (average 4.5 NISP/m^3^). It has been suggested at some other sites that shell was used as pigment processing containers (e.g., [89, 90]), and is more of a consideration for the Ostreidae than the small mangrove gastropods. The isolated oysters occurring below Layer 6 are more likely to be food debris than tools or containers. In addition to being common economic species, these specimens do not bear evidence of ochre or anthropogenic modification. From the terminal Pleistocene (Layers 6 and 5, ≥14 kya), however, economic marine molluscs from rocky or mangrove intertidal habitats (Ostreidae) were increasingly transported to the site (38.2 and 315.5 NISP/m^3^). As this takes place prior to marine incursion into Kilifi Creek (based on the sea level curve in Fig 2), these oysters would have been harvested on the coast and transported at least 15 km inland to PYS. It is also possible that the oysters were processed at the point of acquisition, with the meat removed and preserved, and the shell discarded prior to transportation inland, either carried directly or indirectly via trade networks [91, 92]. As noted by Hausmann et al. [93], even over long distances, the byproducts of field processing (i.e., the shell) should still conform to expectations of transport constraints with shell density decreasing with greater distance from the source [72]. Importantly, the major peak in Ostreidae density in Layer 4 (8 kya, 544.8 NISP/m^3^) corresponds with sea levels approximating modern levels. If suitable mangrove habitats for the establishment of oyster populations [94] kept pace with sea level fluctuations (as suggested further south for the Rufiji Delta and the southwest coast of Zanzibar in Tanzania [95, 96]), during this period people may have been able to access these taxa from within Kilifi Creek, thereby reducing the transport distance to c.8-9 km. Notably, the chert source for Panga ya Saidi’s lithics is located 7 km away from the site in the direction of Kilifi Creek and the highest proportion of chert use is in Layers 5–1 [54], indicating a focus on long-distance resource procurement from this direction.

A change to low amplitude fluctuations following the final phase of late Holocene sea level regression at c.1.2 kya coincides with a taxonomic shift to mangrove habitat gastropods (*Cerithidea* spp.) from Layer 3 into Layer 1 (increasing from 28.2 to 228.1 NISP/m^3^). Mangrove habitat shifts resulting from sea level rise and sedimentation post-stabilisation in the Kilifi Creek Lagoon may have made the exploitation and inland transportation of these small-bodied gastropods more profitable during the late Holocene. Several researchers have noted an approximate maximum distance travelled for the purpose of harvesting shellfish of 10 km [20, 76, 97]. Recently Parkington et al. [92] have suggested 7 km based on a review of mid to late Holocene archaeological data from the Cape west coast of South Africa, correlating with distances of 5–7 km reported from ethnographic records [98, 99]. As PYS lies 15 km from the coast and 8–9 km from Kilifi Creek Lagoon, use of marine resources would have required relatively long-distance transport irrespective of the time period of occupation at the site. Importantly in this context, both the Ostreidae and Potamididae are readily transportable, can stay alive (and therefore fresh) for several days by sealing the shell (e.g., [100]) and do not serve a known symbolic/aesthetic function.

Also of relevance here is the appearance of marine fish in the PYS sequence (Table 2) from Layers 4 to 2 (8 kya to c.1.2 kya) and increasing in Layer 1 (0.5 kya), which corresponds with the increasing discard of the mangrove gastropod *Cerithidea decollata*. These marine fish data include 13 bony fish families that commonly inhabit shallow inshore coastal waters in estuaries and around coral reefs, similar to those Iron Age assemblages reported from the inland sites of Chombo, Mtsengo, and Mbuyuni, located 15 to 25 km from the coast [46]. Interestingly, of the 13 families reported from PYS, a modern assessment of fish diversity and catch location in Kilifi Creek indicates the majority come from closer to the open sea at the mouth of the creek rather than in the middle of the lagoon or into the tidal creeks [51]. Therefore, the harvesting and transportation of the late Holocene molluscan assemblages, when considered relative to the marine fish data, could have resulted from direct acquisition and movement of people using these coastal resources for subsistence. This is an important difference from preceding periods, while the PYS terrestrial faunal and isotopic data suggest broad continuity in foraging through time [37], in line with the lithic evidence [54] the marine mollusc and fish data raises the possibility of greater mobility and more systematic use of coastal resources (e.g., [101]). Again, this suggestion requires further exploration as more data from eastern Africa becomes available.

Freshwater resource exploitation is known to have occurred early in Africa, being associated with early hominins from c.2 mya (summary in [36]). At PYS, the increasing discard of freshwater bivalves (Iridinidae) during the Holocene is important as it coincides with the increase in discard of intertidal marine molluscs. As with other members of the Unionoida, the larvae of these freshwater bivalves are obligate parasites of freshwater fish, with populations requiring ecologically stable waterways [102] connected to a hydrographic network, being found buried in sandy/muddy substrates in running and standing waters with marked seasonal or intermittent level fluctuations [103]. While adapted to relatively dry conditions and resisting desiccation, the Iridinidae appear less able to tolerate increasing saline conditions [104], as would be the case within areas like Kilifi Creek Lagoon. Given the ecological/habitat requirements of these molluscs, this may signal a shift towards the transportation of freshwater resources (whether this was at proximity or greater distance from the site is unclear) or, with the closest perennial river currently being located 5–6 km to the northwest of PYS, could indicate a localised environmental change with these aquatic habitats becoming more available after the Pleistocene. Other freshwater resources are largely absent from the PYS sequence (except for one possible catfish bone in Layer 13 dating to 59 kya, Table 3). The Iridinidae therefore may have been collected logistically alongside freshwater fish, indicating differential use, transportation, and discard of both molluscan and fish resources.

**Table 3:** PYS fish quantification (NISP) by layer.

				Layer						
Class	Order	Family	Taxon	1	2	3	4	13	Total	%
Actinopterygii	Beloniformes	Hemiranphidae	cf. Hemiranphidae			1			1	2.4
	Carangiformes	Carangidae	Carangidae	2					2	4.8
	Labriformes	Labridae	Labridae	1					1	2.3
		Scaridae	Scaridae	3	1				4	9.5
	Mugiliformes	Mugilidae	Mugilidae	1					1	2.4
	Perciformes	Acanthuridae	*Naso* sp.		1				1	2.4
		Haemulidae	*Plectorhincus* sp.	1					1	2.4
		Lethrinidae	*Lethrinus* sp.	6		2			8	19.1
		Lutjanidae	Lutjanidae	1	1				2	4.8
			*Lutjanus* sp.			1			1	2.4
		Serranidae	Serranidae	2		1	1		4	9.5
			*Epinephelus* sp.	1					1	2.4
		Siganidae	*Siganus* sp.	7					7	16.8
		Sparidae/Lethrinidae	Sparidae/Lethrinidae		4				4	9.5
		Sparidae	cf. *Acanthopagrus* sp.		1				1	2.4
	Siluriformes		Siluriformes^a^					1	1	2.4
Chondrichthyes	Carcharhiniformes	Carcharhinidae	Carcharhinidae	2					2	4.8
**Total NISP**				**27**	**8**	**5**	**1**	**1**	**42**	
**NID (not identified)**				**2**	**8**	**1**			**11**	
**Total Weight (g)**				**9.47**	**1.64**	**1.18**	**0.15**	**0.12**	**12.56**	
**Age BP (kya)**				**0.45**		**1.2**	**8**	**58.5**		

Total NISP, the number of specimens unable to be identified (NID), total weight of fish bone (g) and age BP (kya) are provided for each layer/column.

^a^ represents morphologically closer to freshwater catfish (Clariidae) than marine catfish (Ariidae).

The mollusc record at PYS captures consistent pulses of coastal engagement, documenting changes in the acquisition, transport, and discard of aquatic resources. The sequence begins with low-intensity symbolic shell discard, and gradually shifts to the relatively long-distance transport of marine and freshwater resources for consumption, though still in low-densities. Not only is PYS the first eastern African record of its kind, but it is the first consistent mollusc sequence from the Later Pleistocene through the Holocene in Africa. Even though there are no comparative sites in the region, the shifts identified in these data allow us to initially consider the long-term behavioural trends that underpin the archaeological record. The data from PYS help demonstrate that people were present on the coast of eastern Africa from at least 67 kya, that peri-coastal people engaged with marine resources, and that the nature of these engagements changed through time. As noted above, against the dominant terrestrial signals displayed by much of the PYS evidence [37], the molluscan data hints at local and potentially broader changes in population size, trade networks and/or transportation, and landscape mobility through time, and these avenues should be explored by future research. The remarkable record at PYS emphasises the significance of considering peri-coastal/inland sites for understanding the nature of early and long-term marine engagements, something highlighted in syntheses of the MSA African coastal evidence (e.g., [11]) and more broadly (e.g., [105, 106]).

## Conclusion

PYS holds a 67,000 year long stratified record of coastal resource engagement in eastern Africa. As such, PYS data fills important chronological and geographic gaps, enhancing our understanding of marine engagement through MIS 5–1. The molluscan data indicates several phases of coastal interaction with marine and freshwater resources from the Pleistocene through to the Holocene, but also periods with little to no coastal engagement. This mirrors the continent-wide variability in LSA behaviours, perhaps indicating shifts in the resource use of a single population, or the periodic appearance of groups with different land-use strategies. There is a connection between post-glacial sea level rise, coastal reconfiguration and habitat reorganisation, and the changes noted in the taxa discarded at PYS, but these factors cannot fully explain the nature of the shifts seen across the site sequence. In particular, there appear to be marked differences in the nature of engagement with the coast viewed from the aquatic molluscan material, with variable evidence throughout the sequence related to collection and transportation of symbolic materials and the likely dietary contribution of marine gastropods and bivalves. These data potentially signal changes in mobility into the terminal Pleistocene and the Holocene, with people broadening the resources and habitats they exploited with increasing mobility, as well as changes in territoriality and/or demography.

The peri-coastal location of PYS protects it from the periodic flushing or submersion that affects South African caves, but it also reflects mollusc discard choices tied to long-distance transport, rather than the full spectrum of coastal resource use. The PYS mollusc assemblage documents a shifting relationship with aquatic resources, beginning with symbolic shell curation and culminating in systematic low-density marine subsistence resource transportation and discard. This suggests that, even where we do have evidence for early and ongoing coastal engagement, marine resources were more likely to be one component of a broader economic package, reflecting the broad range of options for people at PYS through time. The importance of marine resources is therefore not their absolute contribution in a quantitative sense, but what they potentially add to socio-economic structures when combined with existing strategies for settlement and resource acquisition across a range of habitats. The data from PYS reinforces the notion of complexity and diversity in human behaviour, suggesting that we should not expect coastal or marine engagements to have been a singular advancement. Instead, coastal adaptations are part of a larger package that made our species so flexible in our subsistence behaviour and social lives.

## Supporting information

S1 Fig NISP: MNI scatterplots for the total PYS assemblage by Layer, Ostreidae, Potamididae and Iridinidae (log_10_ transformed). Note differences in horizontal scales.
(TIF)

S2 Fig Species area curve (NTAXA: NISP) for the total PYS assemblage by context. (TIF)

S3 Fig Scatter plot of shell density (NISP/m3) to assemblage richness (NTAXA) by Layer. Terminal Pleistocene-Late Holocene Layers (5–1) filled black circles. Best fit logarithmic trendline used for visual comparison.
(TIF)

S4 Fig Distribution (NISP/m3) through the PYS sequence by Layer of the dominant marine and freshwater taxa. Note differences in horizontal scales.
(TIF)

S1 Table PYS nestedness matrix by context. (XLSX)
