# Supplementary figures and images for: 67,000 years of coastal engagement at Panga ya Saidi, eastern Africa
Source: PLoS One. 2021 Aug 26;16(8):e0256761. doi: 10.1371/journal.pone.0256761 (PMC8389378; doi:10.1371/journal.pone.0256761)

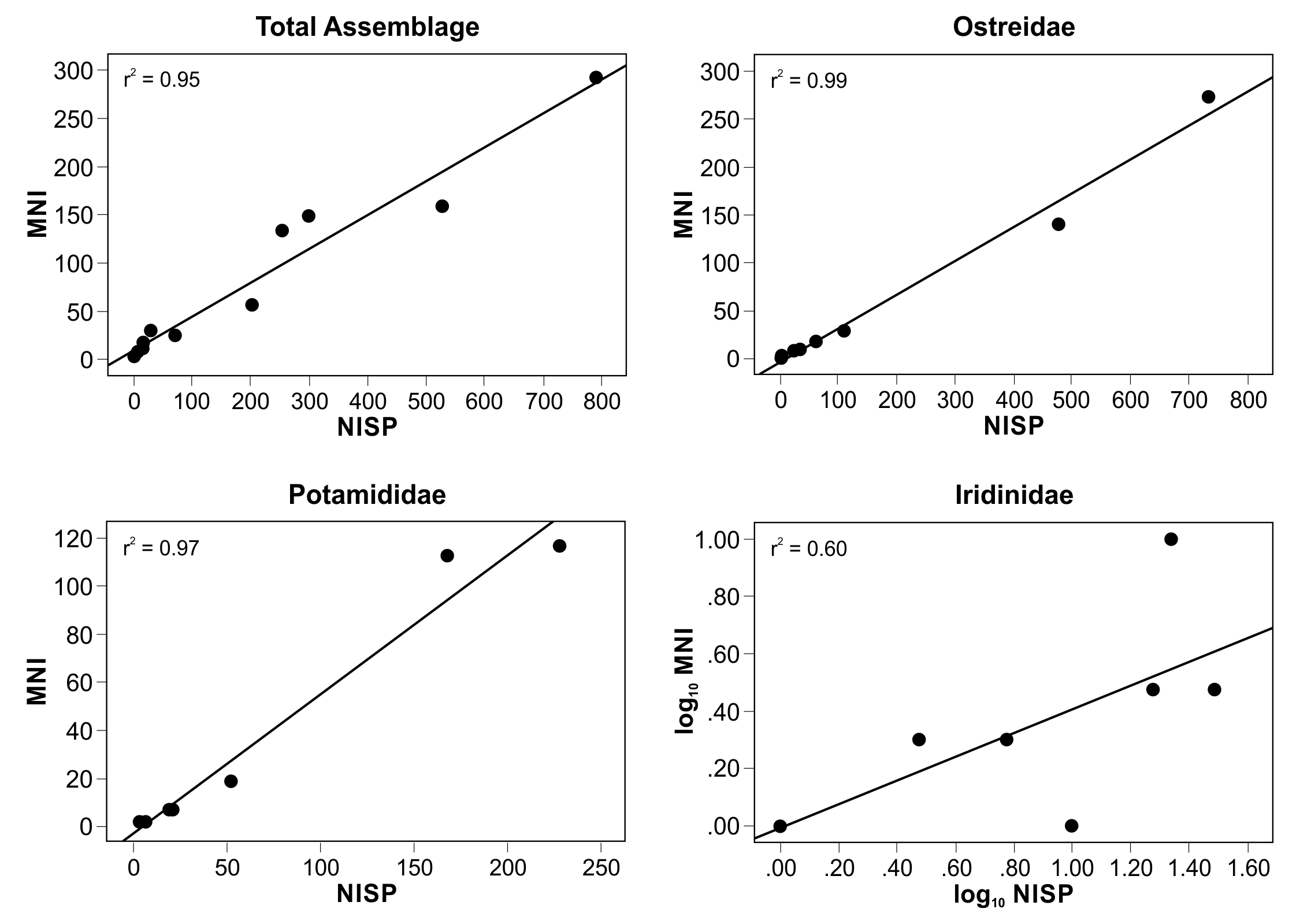

Supplement: S1 Fig — Note differences in horizontal scales. (TIF) [file pone.0256761.s001.tif]

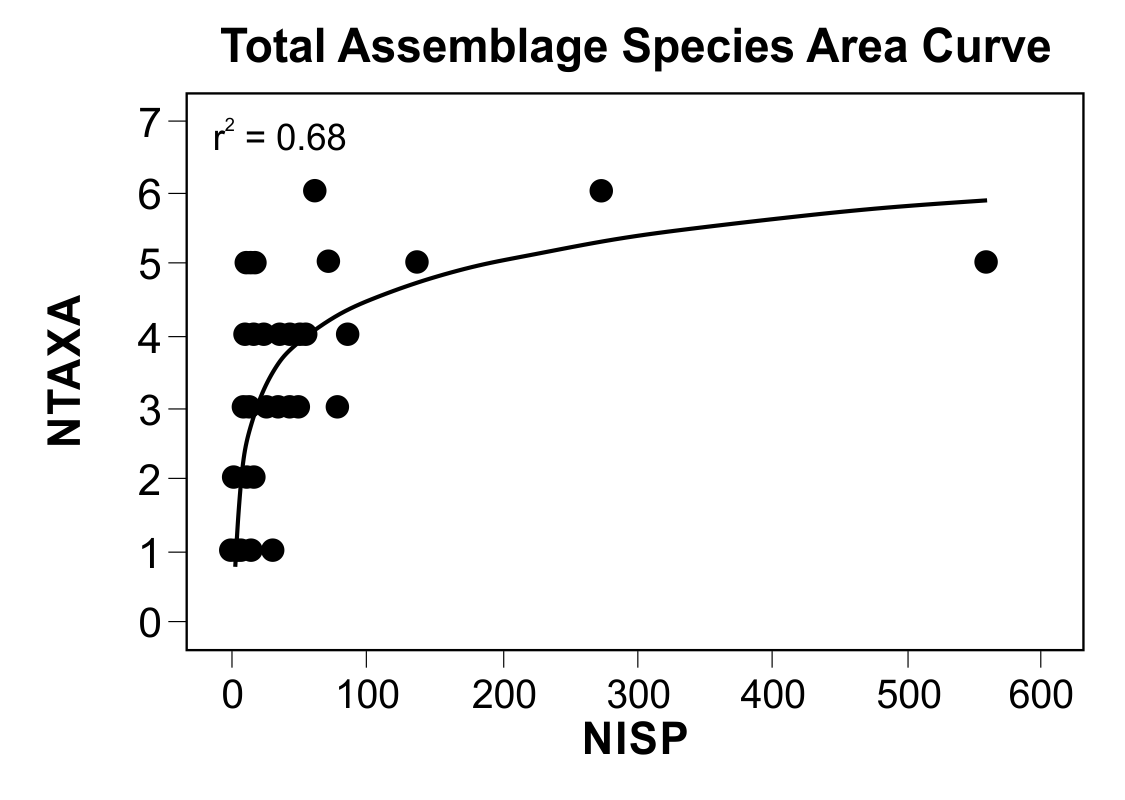

Supplement: S2 Fig — (TIF) [file pone.0256761.s002.tif]

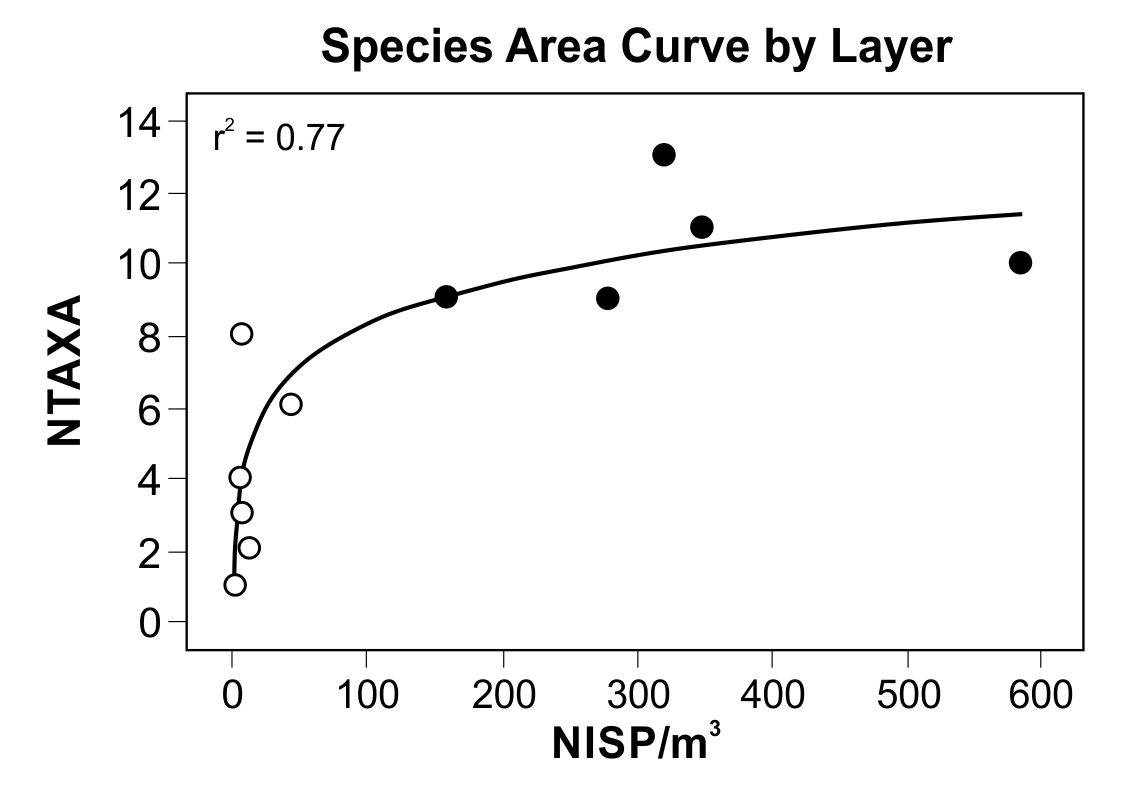

Supplement: S3 Fig — Terminal Pleistocene-Late Holocene Layers (5–1) filled black circles. Best fit logarithmic trendline used for visual comparison. (TIF) [file pone.0256761.s003.tif]

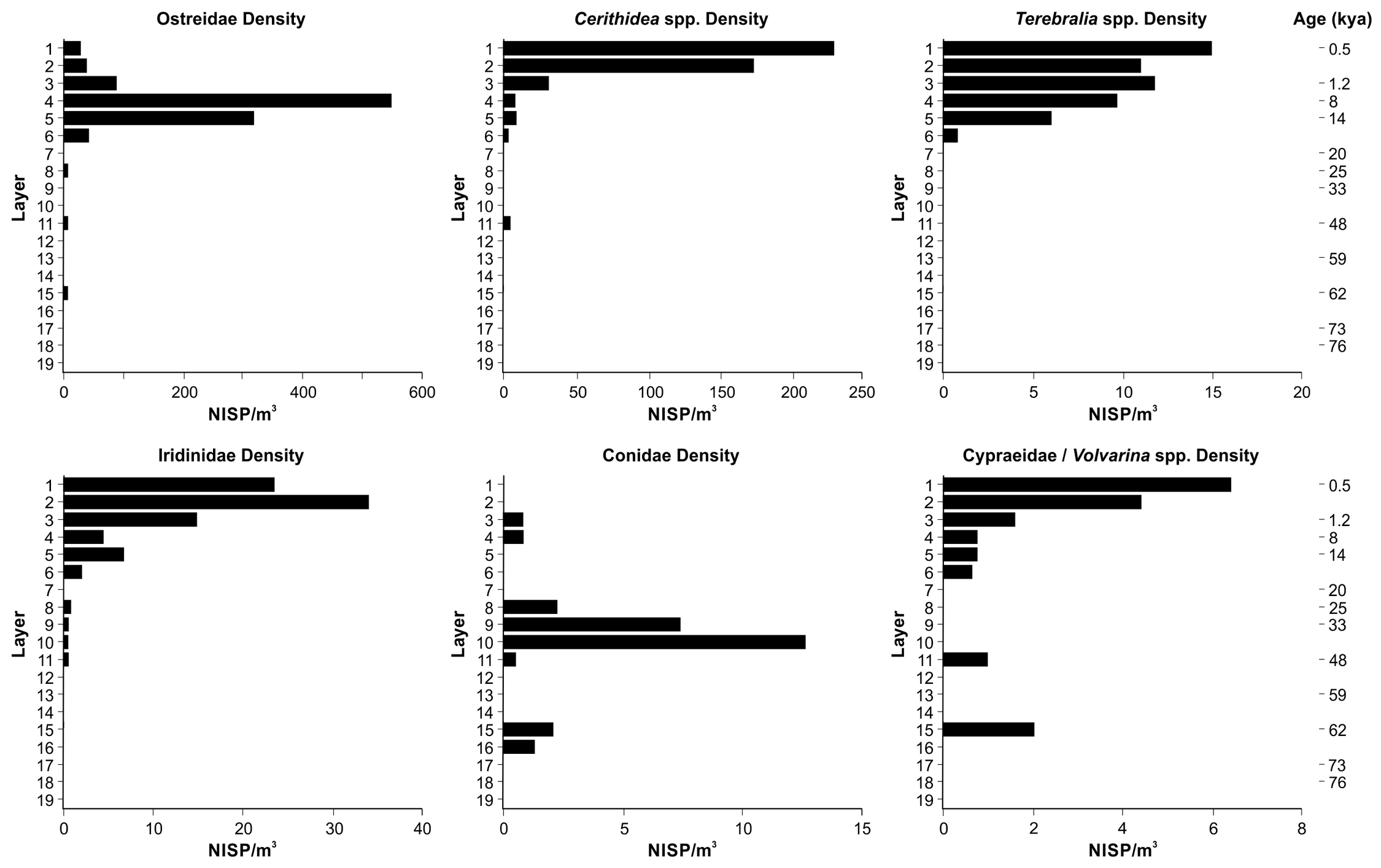

Supplement: S4 Fig — Note differences in horizontal scales. (TIF) [file pone.0256761.s004.tif]
